# Establishment of Human Lung Cancer Organoids Using Small Biopsy and Surgical Tissues

**DOI:** 10.3390/cancers17142291

**Published:** 2025-07-10

**Authors:** Mina Hwang, Junsu Choe, Yong Jae Shin, Bo-Gyeong Seo, Kyung-Mi Park, Sun Hye Shin, Byung Woo Jhun, Hongseok Yoo, Byeong-Ho Jeong, Kyeongman Jeon, Kyungjong Lee, Junghee Lee, Yeong Jeong Jeon, Jong Ho Cho, Seong Yong Park, Hong Kwan Kim, Sang-Won Um

**Affiliations:** 1 Division of Pulmonary and Critical Care Medicine, Department of Medicine, Samsung Medical Center, Sungkyunkwan University School of Medicine, Seoul 06351, Republic of Korea; mn.hwang@sbri.co.kr (M.H.); junsu.choe@samsung.com (J.C.); tjqhrud9@naver.com (B.-G.S.); gyeong.mi@sbri.co.kr (K.-M.P.); fresh.shin@samsung.com (S.H.S.); byungwoo.jhun@samsung.com (B.W.J.); hongseok.yoo@samsung.com (H.Y.); bh82.jeong@samsung.com (B.-H.J.); kjeon@skku.edu (K.J.); kj2011.lee@samsung.com (K.L.); 2 Innovative Institute for Precision Medicine, Samsung Medical Center, Seoul 06351, Republic of Korea; yongjae.shin@samsung.com; 3 Department of Surgery, Samsung Medical Center, Sungkyunkwan University School of Medicine, Seoul 06351, Republic of Korea; 4 Department of Health Sciences and Technology, SAIHST, Sungkyunkwan University, Seoul 03063, Republic of Korea; 5 Department of Thoracic and Cardiovascular Surgery, Samsung Medical Center, Sungkyunkwan University School of Medicine, Seoul 06351, Republic of Korea; jhts.lee@samsung.com (J.L.); ts.yj.jeon@samsung.com (Y.J.J.); jongho9595.cho@samsung.com (J.H.C.); seong.yong.park@samsung.com (S.Y.P.); hkts@skku.edu (H.K.K.)

**Keywords:** co-culture, fibroblasts, immune cells, lung cancer, patient-derived tumor organoid

## Abstract

**Background/Objectives:** Lung cancer is a highly diverse disease, and reliable preclinical models that accurately reflect tumor characteristics are essential for studying lung cancer biology and testing new therapies. This study aimed to establish patient-derived tumor organoids (PDTOs) using small biopsy samples and surgical specimens to create a model system that preserves the genetic and histological features of the original tumors. **Methods:** PDTOs were generated from 163 lung cancer specimens, including 109 samples obtained using endobronchial ultrasound-guided transbronchial needle aspiration (EBUS-TBNA) or bronchoscopy, 52 surgical specimens, and 2 pleural fluid samples. The organoid establishment rate beyond passage three was assessed, and histological subtypes and genetic profiles were analyzed using immunohistochemical staining and targeted exome sequencing. **Results:** The overall PDTO establishment rate was 34.4% (56/163), and 44.6% (25/56) of these organoids retained the histological and genetic features of the parental tumors. Genetic analysis identified key mutations, including KRAS G12C, EGFR L858R, MET exon 14 skipping mutation, and ROS1 fusion. PDTOs successfully formed tumors in mice while maintaining the genetic characteristics of the original tumors. Co-culture of PDTOs with cancer-associated fibroblasts (CAFs) resulted in increased resistance to paclitaxel. In the co-culture model of PDTOs with immune cells, dose-dependent growth inhibition of PDTOs was observed in response to immune checkpoint inhibitors. **Conclusions:** PDTOs established from small biopsy and surgical specimens serve as a valuable model for studying lung cancer biology, tumor microenvironment interactions, and drug response. This model has the potential to improve personalized treatment strategies.

## Simple Summary

Lung cancer is a complex disease with many different forms, making it difficult to study and treat effectively. To improve research and drug development, scientists need reliable models that closely resemble real tumors. In this study, we created patient-derived tumor organoids using small lung biopsy samples and surgical tissues. These organoids successfully expanded in vitro while preserving key features of the original tumors, including their genetic alterations. Some of these organoids were also able to form tumors in mice, allowing additional in vivo evaluation. By using these models, we examined how tumors respond to different treatments, including chemotherapy and immunotherapy. This research provides a valuable tool for studying lung cancer, predicting drug resistance, and developing more effective treatments. Our findings could help researchers better understand the disease and improve personalized treatment strategies for patients.

## 1. Introduction

Lung cancer is a leading cause of death worldwide, with a 5-year survival rate of only 10–20% [1,2]. However, as individuals are genetically diverse, it is difficult to quickly select and administer an appropriate therapy, and tumor heterogeneity can complicate the relationship between genes and drugs [3].

Cell monolayers derived from cancer tissues aid the study of human cancer biology but may not reflect the effects of complex mutations and genetic diversity [4]. Large-scale genomic analyses have revealed that lung cancers differ both phenotypically and genetically, with both inter- and intra-tumoral heterogeneity in play [5,6,7]. Moreover, in the era of immunotherapy, complex biological models that include both cancer and immune cells are essential.

Patient-derived xenografts (PDXs) maintain the original molecular characteristics and molecular heterogeneity of human cancer tissues, and have therefore been used to screen chemotherapeutic agents [8,9]. PDX establishment is expensive and time-consuming, and PDXs cannot be used for high-throughput drug screening [10]. Additionally, PDXs are typically generated in immunodeficient mice, which limits their ability to model interactions between tumor and immune cells. This makes them suboptimal for evaluating immunotherapy responses. Three-dimensional (3D) organoids recapitulate a complex biological environment in vitro [11,12,13], and 3D patient-derived organoids (PDOs) closely mirror organ development and composition. PDOs comprise diverse cell types [13,14,15] and exhibit stem cell-like properties when derived from patient tissues [16,17]. Accordingly, patient-derived tumor organoids (PDTOs) from lung cancer patients represent a valuable platform for evaluating intratumoral heterogeneity and therapeutic responses.

In this study, we established PDTOs from small biopsy samples obtained using endobronchial ultrasound-guided transbronchial needle aspiration (EBUS-TBNA) and surgical specimens on patients with lung cancer. We explored whether the PDTOs aided drug sensitivity testing. PDTOs were co-cultured with fibroblasts and immune cells to investigate chemotherapy resistance and immune checkpoint inhibitors (ICI) responses, respectively.

## 2. Materials and Methods

### 2.1. Human Tissues

Lung tumor tissues and peripheral blood were obtained from patients who underwent EBUS-TBNA or surgery at the Samsung Medical Center between March 2019 and March 2023. The study protocol was approved by the institutional review board of the center (IRB No.: 2014-03-140); all samples and biopsies were collected after obtaining written informed consent from all patients. Fresh samples were placed on ice in advanced Dulbecco’s Modified Eagle Medium (DMEM)/nutrient mixture F12 containing 1× glutamax, 10 μM Y27632, 10 mM HEPES, and PCN/STN/Primocin (adDF+++ medium) and immediately transported to the laboratory. This study was performed in accordance with the Declaration of Helsinki.

### 2.2. Establishment of PDOs

**Figure 1:**
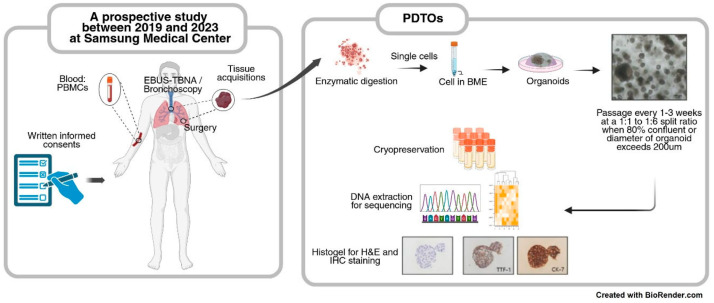
Establishment of patient-derived tumor organoids (PDTOs). EBUS-TBNA, endobronchial ultrasound-guided transbronchial needle aspiration. PBMCs, peripheral blood mononuclear cells. BME, basement membrane extract.

Tumor specimens were obtained from small biopsies performed using EBUS-TBNA or bronchoscopy, as well as from malignant pleural effusions and surgical resections. Tumor tissues were cut into 1–2-mm^3^ pieces using scalpels and digested for 30–60 min in 10 µM Y-27632, 100 µg/mL DNase I, and 500 µg/mL Liberase TH (cat. no. 5401151001; Roche, Basel, Switzerland) in adDF+++ medium, and passed through a sterile 70-μm filter into a 50 mL tube. Digestion was stopped using the addition of 10 mL adDF+++ with 10% (*v*/*v*) fetal bovine serum (FBS). After centrifugation at 1300 rpm for 3 min at room temperature (RT), cells were counted using an automated device. Each pellet was resuspended in 40 μL of ice-cold Cultrex growth factor-reduced 10 mg/mL BME type 2 (cat. no. 3533-010-02; Trevigen, Gaithersburg, MD, USA) and added to one well of a pre-warmed 24-well suspension culture plate. Each drop solidified over 20 min at 37 °C, and the cells were overlaid with complete LO medium consisting of ad-DF+++ supplemented with 10% (*v*/*v*) Noggin conditioned medium, 10% (*v*/*v*) R-spondin-1 conditioned medium, 1× B27 supplement (Gibco, Thermo Fisher Scientific, Waltham, MA, USA), 1.25 mM N-acetylcysteine (Sigma-Aldrich, St. Louis, MO, USA), 10 mM nicotinamide (Sigma-Aldrich), 25 ng/mL human recombinant FGF-7 (Peprotech, Cranbury, NJ, USA), 100 ng/mL human recombinant FGF-10 (Peprotech), 500 nM A83-01 (Tocris Bioscience, Bristol, UK), 1.25 mM hEGF (Gibco), 3 μM CHIR99021 (Tocris Bioscience), and 5 mM Y-27632 (Enzo Life Sciences, Farmingdale, NY, USA). The medium was changed every 3–4 days. Organoids were passaged at a 1:1–1:6 split ratio when the diameter exceeded 200 μm or the confluence attained 80% (Figure 1). PDO establishment was defined as successful organoid culture beyond passage 3. PDTO establishment was defined when the IHC findings were consistent with the tumor histology, and the sequencing results were identical to those of the tumor, or known driver mutations were present if the patient’s tumor sequencing data were unavailable. PDBOs (patient-derived benign organoids) were defined as organoids with IHC staining results inconsistent with tumor histology or sequencing results that did not match the patient’s tumor. Indeterminate organoids were those that failed to yield sufficient cells for IHC staining or sequencing due to passage failure.

### 2.3. PBMC Isolation

PBMCs were isolated using Ficoll-Paque Plus (GE Healthcare Biosciences, Piscataway, NJ, USA) density gradient separation and cryopreserved [18].

### 2.4. IHC Staining of Paraffin-Embedded Organoids

PDOs were recovered from BME using density gradient separation (1200 rpm, 3 min, at RT) when their diameter reached approximately 200 µm. The organoids were then suspended in cold adDF+++ medium, fixed with 4% (*v*/*v*) paraformaldehyde for 30 min at room temperature or overnight at 4 °C, pelleted, embedded in paraffin blocks, sectioned at 5 µm thickness, dewaxed and rehydrated through a graded ethanol series (xylene, 100%, 90%, 75% ethanol, and distilled water), and stained with H&E. IHC staining confirmed the presence of tumor cells. For antigen retrieval, slides were submerged in pre-heated citrate antigen retrieval buffer (10 mM sodium citrate, pH 6.0) and boiled for 15 min, cooled in buffer for 20 min, washed three times under running water (3 min each time), and permeabilized with 0.3% (*v*/*v*) Triton-X in phosphate-buffered saline (PBS) for 15 min. The primary antibodies for IHC were as follows; TTF-1 (cat. no. ab76013; Abcam, Cambridge, UK), CK7 (cat. no. 790-4509; Ventana Medical Systems, Oro Valley, AZ, USA), p63 (cat. no. 790-4509; Ventana Medical Systems), CK5/6 (cat. no. M7237; Dako, Glostrup, Denmark), CD56 (cat. no. 760-4596; Ventana Medical Systems), and synaptophysin (cat. no. ab32127; Abcam). Primary antibodies were diluted at 1:500 and 1:200 in 5% (*v*/*v*) normal donkey serum in PBS and applied to the slides, which were then incubated with secondary antibodies (cat. nos. AI-2000, AI-1000; Vector Laboratories, Newark, CA, USA) at a 1:500 dilution. Visualization was performed using the ultraView Universal DAB Detection Kit (Ventana Medical Systems).

### 2.5. Targeted Exome Sequencing and EGFR Mutation Analyses

Tumor tissues and established organoids were subjected to targeted exome sequencing (CancerSCAN, Tokyo, Japan) [18] or Sanger sequencing to validate the genotypes. If a tumor harbored an *EGFR* mutation, we first subjected established organoids to polymerase chain reaction (PCR) and Sanger sequencing of *EGFR*. If the results were negative, we next performed targeted exome sequencing. If a tumor lacked an *EGFR* mutation, we subjected patient tissues and PDOs to targeted exome sequencing to validate the genotypes. DNA was extracted from PDOs using a QIAamp DNA Mini Kit (cat. no. 51304; Qiagen, Hilden, Germany) following the manufacturer’s protocol. The purified DNA concentration (ng/µL) and the A260/A280 ratio were assessed using a Nanodrop 1000 device (Thermo Fisher Scientific). *EGFR* mutational status was evaluated using 50 ng of genomic DNA that was PCR-amplified in a 20-μL mixture of 10 μL AmpliTaq Gold 360 master mix (cat. no. 4398881; Thermo Fisher Scientific) with 7.6 μL distilled water and 1.4 μL of *EGFR* forward and reverse primer solutions. Amplification proceeded on a thermal cycler at 95 °C for 10 min; followed by 35 cycles of 95 °C for 30 s, 55 °C for 30 s, 72 °C for 60 s, 72 °C for 10 min; and holding at 4 °C. The *EGFR* primers (all 5′ to 3′) were exon 19 (forward: ACATCCACCCAGATCACTGG, reverse: GGAGATGAGCAGGGTCTAGA), exon 20 (forward: ATTCATGCGTCTTCACCTG, reverse: CAGACCGCATGTGAGGATC), and exon 21 (forward: TGAATTCGGATGCAGAGCT, reverse: CTGCGAGCTCACCCAGAAT). All PCR products were verified using electrophoresis on agarose gels. The PCR products were sequenced and analyzed using the Bioneer Corporation (Seoul, Republic of Korea). Sequencing reactions were performed in both the forward and reverse directions, and all mutations were confirmed by sequencing the individual PCR products.

### 2.6. Xenograft Establishment Using PDTOs

PDTOs were implanted into 4-to-5-week-old male nude (nu/nu) mice from Orient Bio (Seongnam, Republic of Korea). PDTOs were harvested at volumes > 200 mm^3^, and suspensions (5 × 10^5^ cells) in 50% Matrigel/50% LO medium (both *v*/*v*) were subcutaneously injected into mouse flanks using a 30-G needle. The tumor size was measured weekly using calipers. The average tumor volume in each group (mm^3^) was calculated using the equation for a prolate spheroid: 0.523 × (large diameter) × (small diameter) [19]. Tumors were measured and photographed weekly. Mice were weighed at treatment commencement, then weekly, and at the end of treatment. Throughout the study, we confirmed that there were no significant or rapid changes in body weight. Xenograft tumors were harvested after 5 weeks of drug treatment. All methods complied with the guidelines of our Institutional Animal Care and Use Committee (IACUC) as approved by the Association for Assessment and Accreditation of Laboratory Animal Care (AAALAC). All animal studies were performed in accordance with the Animal Research Reporting of In Vivo (ARRIVE) guidelines.

### 2.7. Culture of PDX Cells

Xenograft tumors approximately 300 mm^3^ in volume were dissected out, finely minced, placed in 15 mL Falcon tubes with Liberase TH (cat. no. 5401151001; Roche), and incubated at 37 °C in a water bath. The digested tissue was filtered through a 40 µm cell strainer and washed with advanced DMEM/F12 medium (cat. no. 11320033; Gibco). The cells were collected, and FBS was added to 20% (*v*/*v*) to neutralize enzymes. Each mixture was centrifuged at 1300 rpm for 3 min, and the xenograft cell pellet was resuspended in adDF+++ medium that was changed every 3 to 4 days.

### 2.8. High-Throughput Drug Screening Using PDTOs

PDTOs were collected and plated to analyze drug responses when PDTO diameters attained 200 μm. PDTOs were resuspended in 70% (*v*/*v*) BME/LO culture medium and dispensed in duplicate into ultralow-attachment 384-well plates (Corning Life Sciences, Corning, NY, USA) and cultured for 5 days. PDTOs were subsequently treated with cisplatin, irinotecan, pemetrexed, crizotinib, brigatinib, ceritinib, carboplatin, paclitaxel, gemcitabine, alectinib, lorlatinib, gefitinib, etoposide, docetaxel, vinorelbine, erlotinib, afatinib, osimertinib, and sotorasib (all from Selleckchem, Houston, TX, USA). Media were aspirated from the wells, and drug solutions in fresh media were added. After 5 days of incubation, PDTO viability was assessed using the CellTiter-Glo 3D (G968B; Promega, Madison, WI, USA) method. At the indicated time points, half of the medium (100 μL) was removed from each well and 100 μL of ATP assay medium was added, followed by incubation at RT with shaking for 30 min. Each supernatant was transferred to a white polystyrene 96-well Costar assay plate (cat. no. 3912; Corning Life Sciences) and fluorescence was quantified using a Veritas Microplate Luminometer (Turner Biosystems, Sunnyvale, CA, USA).

### 2.9. GFP Lentivirus Transfection of PDTOs

Single organoid cells were prepared as described above for PDTO drug response screening. To compare PDTO growth rates, 1 μg of GFP Lentivirus (cat. no. 631982; TaKaRa Bio, Kusatsu, Japan) was mixed with 6 μL TransIT-Lenti Transfection Reagent (cat. no. MIR6604; Mirus Bio, Madison, WI, USA) according to the manufacturer’s instructions. The reaction mixture (200 μL) was added to 2 × 10^5^ single PDTO cells in a single well of a 24-well plate, incubated at 37 °C for 1 h, and the cells were harvested, pelleted, and seeded in BME into two wells of a 24-well plate, and grown in LO culture medium before co-culture. Western blotting using an anti-GFP antibody was employed to measure GFP expression in transfected PDTOs; GAPDH was used as the internal control. Quantification was conducted through image analysis using Image J software (version 1.54g).

### 2.10. Co-Culture of PDTOs with CAFs and Immune Cells

PDTOs were digested into single cells as described above. During co-culture, patient-matched PDTOs and PBMCs were stained with CellTracker Dye Blue and Red, respectively (cat. nos. Q25049 and Q25029; Invitrogen, Carlsbad, CA, USA). CAF isolation and culture were described in our previous study [18]. Patient-matched PDTOs and CAFs (or PBMCs) were mixed in a 1:1 (or 1:2) ratio and seeded into 96-well plates as 10-μL drops in a co-culture matrix, followed by culture in 100 μL LO. The effects of CAFs on chemotherapy resistance were explored using paclitaxel (cat. no. S1150; Selleckchem). To evaluate immunotherapeutic responses, PDTOs and PBMCs were co-cultured with pembrolizumab (cat. no. A2005; Selleckchem), nivolumab (cat. no. A2002; Selleckchem), atezolizumab (cat. no. A2004; Selleckchem), and an IgG4 isotype control (cat. no. 403701; BioLegend, San Diego, CA, USA) for 7 days. Cells were microscopically examined at similar positions 0 and 7 days after drug application. Image processing and drug response analysis were conducted using a CellTiter-Glo 3D (G968B; Promega) viability assay.

### 2.11. Statistical Analysis

Data are presented as numbers (%) or as means ± standard deviation. Categorical data were compared using Fisher’s exact test. All statistical analyses were performed using SPSS v24 software (IBM Corp., Armonk, NY, USA). Statistical significance was evaluated at a threshold of *p* < 0.05.

### 2.12. Data Availability

All data generated or analyzed during this study are contained within the article and Supplementary Material.

## 3. Results

### 3.1. Clinical Characteristics of the Study Subjects

This prospective study included 163 patients with lung cancer (Figure 1), of whom 128 were males (78.5%). The mean age was 64 years (Table 1). EBUS-TBNA was used to acquire tissues from metastatic lymph nodes (100 patients, 61.3%). Other tissues were obtained during surgery to treat the primary tumor (52 patients, 31.9%); using bronchoscopy (forceps biopsy or transbronchial lung biopsy) or EBUS-TBNA of the primary tumors (9 patients, 5.5%); and using thoracentesis of malignant pleural effusions (2 patients, 1.2%) (Table 1). The most common histological diagnosis was adenocarcinoma (97 patients, 59.5%); a total of 121 (74.2%) patients were of stage III and IV (Table 1).

### 3.2. Establishment of PDOs and PDTOs

Table 2 shows the establishment rates of PDOs, PDTOs, and patient-derived benign organoids (PDBOs); the overall establishment rate of PDOs was 34.4% (56/163). Twenty-five (15.3%) PDOs were confirmed to be PDTOs using immunohistochemical (IHC) staining and targeted exome or Sanger sequencing. Twenty-five (15.3%) PDOs were classified as PDBOs; IHC staining results did not reveal a tumor histology (N = 7), or the sequencing results differed from those of tumor tissues (N = 18). Six (3.6%) PDOs were classified as indeterminate because of passage failure; it was impossible to obtain sufficient cells for IHC staining or sequencing (Table 2).

Figure S1 shows representative light microscopy (LM) images of PDTOs after hematoxylin and eosin (H&E) and IHC staining. The PDTOs were positive for subtype-specific markers; i.e., TTF-1 and CK-7 for adenocarcinomas, p63 and CK5/6 for squamous cell carcinomas, and CD56 and synaptophysin for small-cell lung cancer [14]. The H&E staining results indicated similar morphologies between PDTOs and patient tumor tissues (Figure S1). Thus, the original tissue characteristics were maintained in the organoids. Next, we used targeted exome sequencing to explore whether the PDTOs retained patient-specific tumor genetic alterations. The genetic characteristics of the 26 PDTOs, consistent with those of the original tumor tissues, are summarized in Tables S1 and S2. Of the 26 PTDOs, 13 were established from specimens collected from metastatic lymph nodes or primary lesions using EBUS-TBNA. The PDTOs harbored known driver mutations, including *KRAS* G12C, *EGFR* L858R, the *MET* exon 14 skipping mutation, the *ERBB2* mutation, *ROS1* fusion, etc.

Figure S2 shows representative LM H&E and IHC staining results for PDBOs. The PDBOs were negative or weakly positive for histological subtype markers upon IHC staining (Figure S2).

### 3.3. Factors Associated with Successful PDTOs Establishment

Next, we sought factors conducive to successful PDTO establishment (Table 3). The establishment rate did not differ by histological subtype (*p* = 0.976). The highest establishment rate was observed in samples obtained from primary tumors using bronchoscopy or EBUS-TBNA (33.3%), followed by surgical specimens from primary tumors (23.1%), EBUS-TBNA of metastatic lymph nodes (10.0%), and thoracentesis of malignant pleural effusions (0%) (*p* = 0.060). In terms of genotype, the establishment rate was lower for samples with EGFR mutation (12.9%) compared to those with other genetic alterations (34.4%) (*p* = 0.046).

### 3.4. In Vivo Tumorigenesis Assays of PDTOs Using Patient-Derived Xenografts (PDXs)

**Figure 2:**
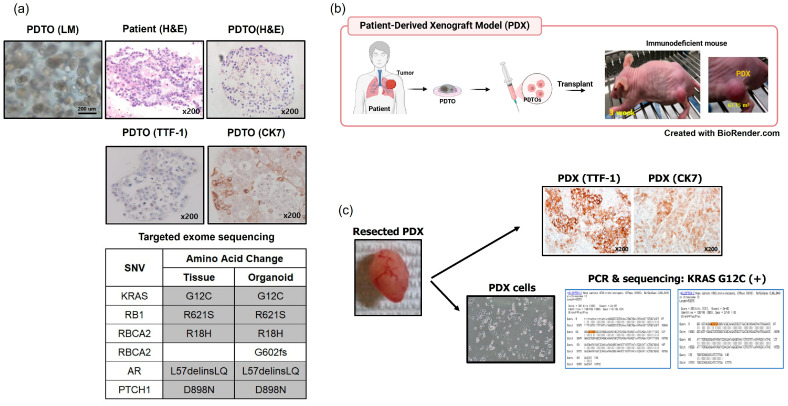
Results of an in vivo tumorigenesis assay using a TH81 PDTO derived from a lung adenocarcinoma harboring a KRAS G12C mutation. (**a**) The TH81 PDTO and patient tissue were subjected to hematoxylin and eosin (H&E) and immunohistochemistry (IHC) staining, and the histological features were recorded. Targeted exome sequencing confirmed that the TH81 PDTO maintained the genotype of the parental tumor. (**b**) Patient-derived xenografts (PDXs) were generated using subcutaneous injection of TH81 PDTOs into nude mice. (**c**) The resected PDX tumors maintained the IHC features of the parental tumor and harbored the KRAS G12C mutation. LM, light microscopy.

We performed in vivo tumorigenesis assays using TH81 PDTO (an adenocarcinoma with the *KRAC* G12 mutation). To prepare PDXs, the TH81 PDTO and patient tumor tissue were subjected to H&E and IHC staining and targeted exome sequencing. The TH81 PDTO maintained the original histological and genetic features (Figure 2a). A schematic of TH81 PDTO-derived PDX development in nude mice is shown in Figure 2b. TH81 PDX cells maintained the histological and genetic features of TH81 PDTO, as confirmed with H&E and IHC staining and sequencing (Figure 2b). Thus, the original tumor characteristics were maintained in the PDX.

### 3.5. PDTO-Based High-Throughput Drug Screening

**Figure 3:**
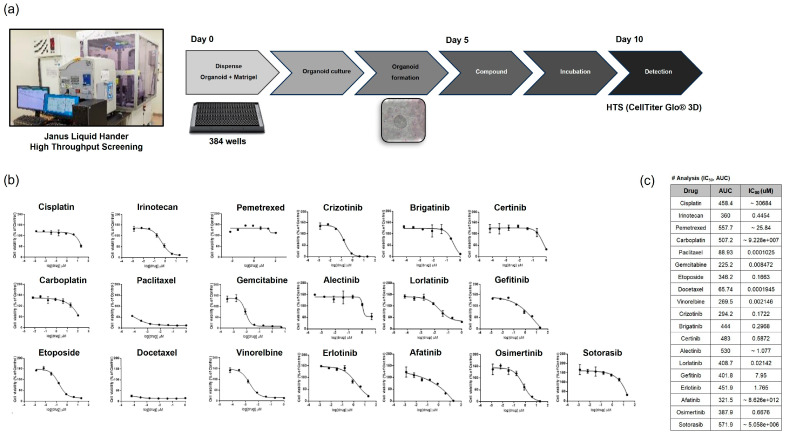
Drug high-throughput screening (HTS) using PDTOs. (**a**) The drug HTS method and timeline were used to test the responses of established PDTOs to chemotherapeutic agents. Representative (**b**) drug response curves and (**c**) half-maximal inhibitory concentration (IC50) values for 19 drugs derived using TH224 PDTOs.

We used the PDTOs to conduct high-throughput screening of chemotherapeutic agents (Figure 3). The methods and timeline are summarized in Figure 3a. After 5 days of organoid formation, 5 days of drug treatment followed. Figure 3b,c show representative drug response curves and half-maximal inhibitory concentration (IC_50_) values for 19 drugs where the TH224 PDTO. Drug sensitivity data were available within 10 days.

### 3.6. Co-Culture of PDTOs and Cancer-Associated Fibroblasts (CAFs) or Immune Cells

**Figure 4:**
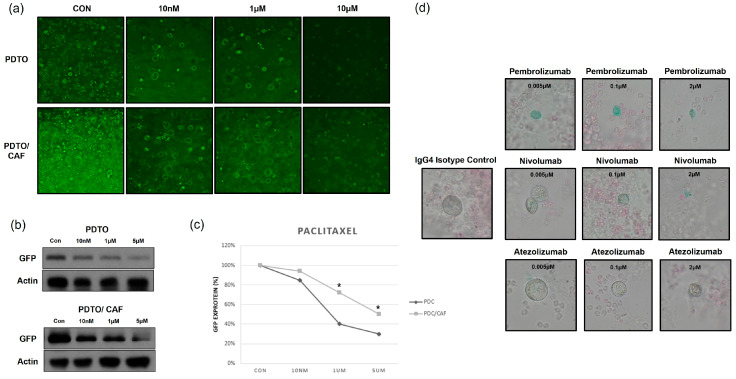
Co-culture of PDTOs with CAFs and immune cells. (**a**) Green fluorescence protein (GFP) immunofluorescence images of direct co-cultures of patient-matched PDTOs (TH122) and CAFs growing in three concentrations of paclitaxel (10 nM, 1 uM, and 5 uM). (**b**) Western blotting using a GFP-specific antibody revealed GFP expression by transfected PDTOs; glyceralde-hyde-3-phosphate dehydrogenase (GAPDH) was used as the internal control. (**c**) GFP quantification using image analysis employing Image J software. Data are means ± standard deviation (* *p* < 0.01). (**d**) Patient-matched PDTOs and PBMCs were stained using CellTracker Dye Blue and Red, respectively. PDTOs and PBMCs were co-cultured with three concentrations of immune checkpoint inhibitors (ICIs; pembrolizumab, nivolumab, and atezolizumab), or an IgG4 isotype control, for 7 days. The uncropped bolts are shown in Supplementary Figure S3.

We co-cultured PDTOs (TH122) with fibroblasts and immune cells to investigate chemotherapy resistance and the response to ICIs (Figure 4). PDTOs expressing green fluorescent protein (GFP) were generated using lentiviral transduction (Figure 4a). Patient-matched PDTOs and CAFs were co-cultured using the basement membrane extract (BME) droplet method to emulate the extracellular matrix. After 7 days of paclitaxel treatment, fluorescence microscopy (Figure 4a), Western blotting of GFP (Figure 4b and Figure S3), and GFP quantification (Figure 4c) demonstrated that PDTOs co-cultured with CAFs exhibited increased resistance to paclitaxel chemotherapy compared to PDTOs cultured alone.

Next, we co-culture PDTOs and peripheral blood mononuclear cells (PBMCs). The parental tumor of TH122 demonstrated 90% PD-L1 positivity by IHC using the 22C3 clone, and the established PDTO also exhibited PD-L1 expression, as confirmed with IHC (Figure S4). Patient-matched PDTOs and PBMCs were stained using blue and red CellTracker Dye, respectively (Figure 4b). PDTOs and PBMCs were co-cultured with three concentrations of each of three ICIs, pembrolizumab, nivolumab, and atezolizumab; the control was an IgG4 isotype. After 7 days of co-culture, PDTO growth was observed using bright-field microscopy. As the concentrations of ICIs increased, organoid growth declined (Figure 4d).

## 4. Discussion

We established PDTOs using small biopsy specimens obtained using EBUS-TBNA, bronchoscopy, and surgical resection from patients with primary lung cancer. The overall establishment rates for PDOs and PDTOs were 34.4% and 15.3%, respectively. PDTOs successfully grew into tumors in BALB/C nude mice, and the PDXs maintained the genotypes of the parental tumors. The PDTOs were subjected to high-throughput drug screening and co-cultured with fibroblasts and immune cells, yielding data on chemotherapy resistance and ICI response, respectively.

PDTO establishment rates vary significantly by tumor type. High success rates (>75%) have been reported for breast, colorectal, and pancreatic cancer [20,21,22], whereas prostate, liver, and esophageal cancers show lower rates (15–50%) [23,24,25]. The success rates for primary lung cancers range from 7–88% [26,27,28,29,30,31], perhaps influenced by tumor genetic backgrounds, differences in the culture media employed, or definitions of PDTO establishment. In this study, we defined PDTO establishment very strictly; the IHC findings must be consistent with the tumor histology, and sequencing must yield results identical to those from patient tumors or, if extensive patient sequence data were not available, the PDTOs must harbor the known driver mutations. Of the 56 successfully established PDOs, 25 (44.6%), 25 (44.6%), and 6 (10.7%) were PDTOs, PDBOs, and indeterminate organoids, respectively. Although we obtained tumor tissues from both primary lesions and metastatic lymph nodes, many PDOs were overgrown by normal epithelial cells. Dijkstra KK et al. also reported the low establishment rate of PDTOs in primary lung cancer (17%) [32]. The authors performed very robust validation for established PDOs using IHC staining, copy number profiling, and Sanger sequencing, and they also found that a significant amount of PDOs were overgrown by normal airway organoids. The PDTO model could be improved by optimizing culture conditions and incorporating genotype-specific growth factors to enhance tumor selectivity and reduce normal cell overgrowth [28,33]. Applying single-cell RNA sequencing and lineage tracing would enable precise distinction between tumor and non-tumor cells within organoid cultures [21]. These refinements would increase the model’s reliability for patient stratification, drug testing, and integration into clinical trial design [34].

We found that PDTO establishment rates did not differ markedly by histological subtypes and tissue acquisition methods (Table 3). However, tumors harboring EGFR mutations showed a significantly lower PDTO establishment rate (12.9%) compared to those with other genetic alterations (34.4%) (*p* = 0.046; Table 3). In a recent study, Togasaki et al. successfully established 39 lung cancer organoid lines from 31 patients with EGFR-mutant lung cancer, although they did not report the establishment rate of PDTOs [35]. They utilized a distinct organoid culture medium containing IGF-1 and 5% Afamin-Wnt-3A serum-free conditioned medium. Further studies are needed to determine the optimal culture conditions for each genetic subtype (e.g., EGFR, KRAS, ALK). In our study, in terms of tissue acquisition methods, primary tumor bronchoscopy or EBUS-TBNA tended to be associated with a higher establishment rate (33.3%) than other methods, and tissue specimens were obtained using bronchoscopy or EBUS-TBNA for 13 (52.0%) of the 25 established PDTOs (Table 3). Park et al. reported a 40.7% success rate in organoid establishment using cryobiopsy during bronchoscopy in a cohort of 113 lung cancer patients [36]. Vigelm et al. also demonstrated successful tumor organoid generation using fine-needle aspiration across various tumor types [37]. The landscape of lung cancer organoid establishment is expanding beyond surgical specimens to include diverse biopsy techniques such as forceps biopsy using bronchoscopy, EBUS-TBNA, cryobiopsy, and FNA.

We used an in-house lung organoid (LO) medium, which is a modification of airway organoid medium [27]. Compared to the airway organoid medium, LO contains CHIR99021 and EGF but not SB202190 (a p38 inhibitor). Various culture media are currently used for primary lung cancer organoid culture [38]. Further refinement of culture conditions, including the standardization of tissue processing and the culture medium, is needed to improve successful PDTO establishment; it is essential to inhibit overgrowth by normal epithelial cells.

The tumorigenesis of established PDTOs was verified using a xenograft model in nude mice. IHC staining and targeted exome sequencing confirmed that the histological subtype (adenocarcinoma) and the genotype (*KRAS* G12C mutation) were maintained in the xenograft and the PDX (Figure 2). PDTOs were used for high-throughput screening of chemotherapeutic agents; the obtained IC_50_ values of 19 agents were available within 10 days. Previous studies have also employed lung cancer PDTOs for drug screening [39,40]. PDTOs from small biopsy specimens will greatly aid precision medicine when treating patients with advanced lung cancer.

The tumor microenvironment includes vascular structures, the extracellular matrix, stromal cells, and immune cells such as lymphocytes, macrophages, and natural killer cells [41,42]. In this study, CAFs increased paclitaxel resistance (Figure 4a–c). Long et al. [43] reported that CAFs increased the cisplatin resistance of bladder cancer cells, possibly using the IL-6/TGF-β pathway and GPR77 signaling [44]. In the co-culture of PDTOs and PBMCs with ICIs (pembrolizumab, nivolumab, and atezolizumab), the ICIs inhibited PDTO growth in a concentration-dependent manner, and there was no significant difference in the response in relation to specific ICIs (Figure 4d). Forsythe et al. [45] similarly used organoid/immune cell co-culture to investigate the efficacy of pembrolizumab against appendiceal cancer.

This study had several limitations. First, the relatively low establishment rate of PDTOs, partly due to overgrowth by normal epithelial cells, limits their broad applicability. Second, the use of a single in-house culture medium may not be optimal for all genetic subtypes. Third, the current co-culture system cannot fully replicate the complexity of the in vivo tumor microenvironment. Although we established a co-culture model of PDTOs with immune cells to evaluate responses to ICIs, the current analysis was primarily limited to assessing organoid viability. We did not evaluate immune-related functional markers, such as cytokine secretion (e.g., IFN-γ, TNF-α) or T-cell activation markers (e.g., CD69, PD-1, granzyme B), which are important for understanding the mechanisms underlying response or resistance to ICIs. Finally, in the co-culture experiment, we used unfractionated PBMCs. Further immunophenotyping (e.g., flow cytometry for CD8^+^ T cells or NK cells) of PBMCs will be necessary in future experiments.

This study shows that lung cancer PDTOs preserve tumor-specific genetic and histological features, enabling more accurate patient stratification based on molecular profiles. The retention of driver mutations such as EGFR L858R and KRAS G12C in PDTOs confirms their genomic fidelity and supports their application in preclinical drug testing, including targeted therapy and chemotherapy, which supports the use of PDTOs in personalized treatment planning [33]. Moreover, the observed chemoresistance in co-culture with CAFs suggests a relevant model to study the tumor–stroma interactions that contribute to treatment failure [41]. Although the PBMC co-culture experiments primarily assessed viability, the observed dose-dependent inhibition by immune checkpoint inhibitors suggests potential for immunotherapy response prediction, even at a qualitative level [46]. These findings underscore the translational relevance of the PDTO model for evaluating both tumor-intrinsic and microenvironment-mediated therapeutic responses. Furthermore, the integration of PDTO-based functional assays into clinical trial design could enhance biomarker-driven patient selection and improve therapeutic outcomes [34].

Future directions for this model include the incorporation of expanded or engineered immune cells, such as CAR-T cells, to evaluate antigen-specific responses and immunotherapeutic efficacy [46]. Integration of CRISPR-based gene editing could enable functional studies of therapy resistance mechanisms by selectively modifying tumor-intrinsic or stromal genes [47]. These approaches would further enhance the utility of the PDTO platform for mechanistic studies and the development of personalized therapeutic strategies [34].

## 5. Conclusions

In conclusion, PDTOs from lung cancer tissues obtained using small biopsies and during surgery can aid the investigation of tumor biology and drug sensitivity and improve the efficacy of personalized immunotherapy.

## Figures and Tables

**Table 1:** Baseline characteristics of study subjects (N = 163).

Characteristics	Number (%) or Mean ± SD
**Age, years**	64.0 ± 9.3
**Male sex**	128 (78.5)
**Method used to acquire tissue specimens**	
EBUS-TBNA of metastatic lymph nodes	100 (61.3)
Surgery on the primary tumor	52 (31.9)
Bronchoscopy or EBUS-TBNA of the primary tumor	9 (5.5)
Thoracentesis of malignant pleural effusions	2 (1.2)
**Histology**	
Adenocarcinoma	97 (59.5)
Squamous cell carcinoma	36 (22.1)
Other NSCLC	4 (1.2)
SCLC	26 (15.9)
**Stage**	
I	24 (14.7)
II	18 (11.0)
III	49 (30.0)
IV	72 (44.2)

EBUS-TBNA, endobronchial ultrasound-guided transbronchial needle aspiration; NSCLC, non-small-cell lung cancer; SCLC, small-cell lung cancer; SD, standard deviation.

**Table 2:** Establishment rates of PDOs and PDTOs (N = 163).

Establishment Rate	N (%)
**PDOs**	**56 (34.4%)**
**PDTOs**	**25 (15.3%)**
All confirmed with IHC staining and sequencing	
**PDBOs**	**25 (15.3%)**
IHC staining results not compatible with the tumor histology	7 (4.3%)
Sequencing results not compatible with patients’ tumors	18 (11.1%)
**Indeterminate organoids**	**6 (3.6%)**
Passage Failure; it was not possible to obtain sufficient cells for	
IHC staining or sequencing	

PDO, patient-derived organoid; PDTO, patient-derived tumor organoid; PDBO, patient-derived benign organoid; IHC, immunohistochemistry.

**Table 3:** Successful establishment of PDTOs according to histology, tissue acquisition methods, and genotypes.

Characteristics	Number (%)	*p*-Value
**Histology**		0.976
Adenocarcinoma (N = 97)	16 (16.4)	
Squamous cell carcinoma (N = 36)	5 (13.9)	
Other NSCLC (N = 4)	0 (0)	
SCLC (N = 26)	4 (15.4)	
**Tissue acquisition methods**		0.060
EBUS-TBNA of metastatic lymph nodes (N = 100)	10 (10.0)	
Bronchoscopy or EBUS-TBNA of primary tumor (N = 9)	3 (33.3)	
Surgery on primary tumor (N = 52)	12 (23.1)	
Thoracentesis of malignant pleural effusions (N = 2)	0 (0)	
**Genotypes**		0.046
EGFR mutation (L858R, exon 19 del, exon 20 insertion, T790M) (+) (N = 31)	4 (12.9)	
Other genetic alterations (N = 61)	21 (34.4)	

ADC, adenocarcinoma; SqCC, squamous cell carcinoma; NSCLC, non-small-cell lung cancer; SCLC, small-cell lung cancer; LN, lymph node; EBUS-TBNA, endobronchial ultrasound-guided transbronchial needle aspiration.

## Data Availability

All data generated or analyzed during this study are contained within the article and Supplementary Material.

## Supplementary Materials

The following supporting information can be downloaded at: https://www.mdpi.com/article/10.3390/cancers17142291/s1, Figure S1: The representative results for LM (scale bar, 200 μm), H&E (magnification, ×200), and IHC (scale bar, 200 μm) staining of PDTOs compared with H&E staining of patients’ tumors. LM, light microscopy H&E, hematoxylin and eosin; IHC, immunohistochemistry; PDTOs, patient-derived tumor organoids; ADC, adenocarcinoma; SqCC, squamous cell carcinoma; SCLC, small cell lung cancer; Figure S2: The representative results for LM (scale bar, 200 μm), H&E (magnification, ×200), and IHC (scale bar, 200 μm) staining of PDBOs compared with H&E staining of patients’ tumors. LM, light microscopy; H&E, hematoxylin and eosin; IHC, immunohistochemistry; PDBOs, patient-derived benign organoids; SqCC, squamous cell carcinoma; SCLC, small cell lung cancer; Figure S3: The original western blot image for Figure 4B. PDTOs were cultured alone or co-cultured with CAFs and treated with increasing concentrations of paclitaxel (0, 10 nM, 1 μM, 5 μM). The untreated group (0 μM) was used as the control (Con). Figure S4. Representative IHC staining results of PDTOs (magnification, ×200). PD-L1 (22C3), Dako Agilent. Table S1. Characteristics of successfully established PDTOs (N = 25). Table S2. Targeted exome sequencing for tumor tissues and organoids.
