# Supplementary material for: Establishment of Human Lung Cancer Organoids Using Small Biopsy and Surgical Tissues
Source: Cancers (Basel). 2025 Jul 10;17(14):2291. doi: 10.3390/cancers17142291 (PMC12293982; doi:10.3390/cancers17142291)
Supplement: Supplementary file 1 [file cancers-17-02291-s001.zip › cancers-3684466-supplementary.pdf]

Supplementary Materials

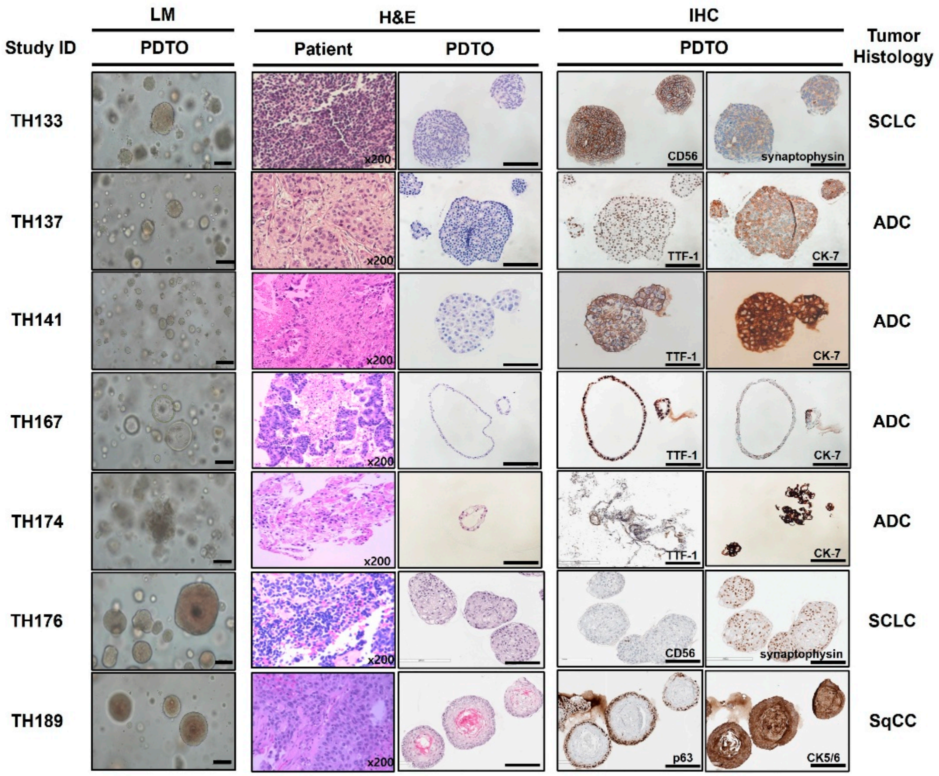

Figure S1. The representative results for LM (scale bar, 200 μm), H&E (magnification, x200), and IHC (scale bar, 200 μm) staining of PDTOs compared with H&E staining of patients' tumors. LM, light microscopy H&E, hematoxylin and eosin; IHC, immunohistochemistry; PDTOs, patient-derived tumor organoids; ADC, adenocarcinoma; SqCC, squamous cell carcinoma; SCLC, small cell lung cancer.

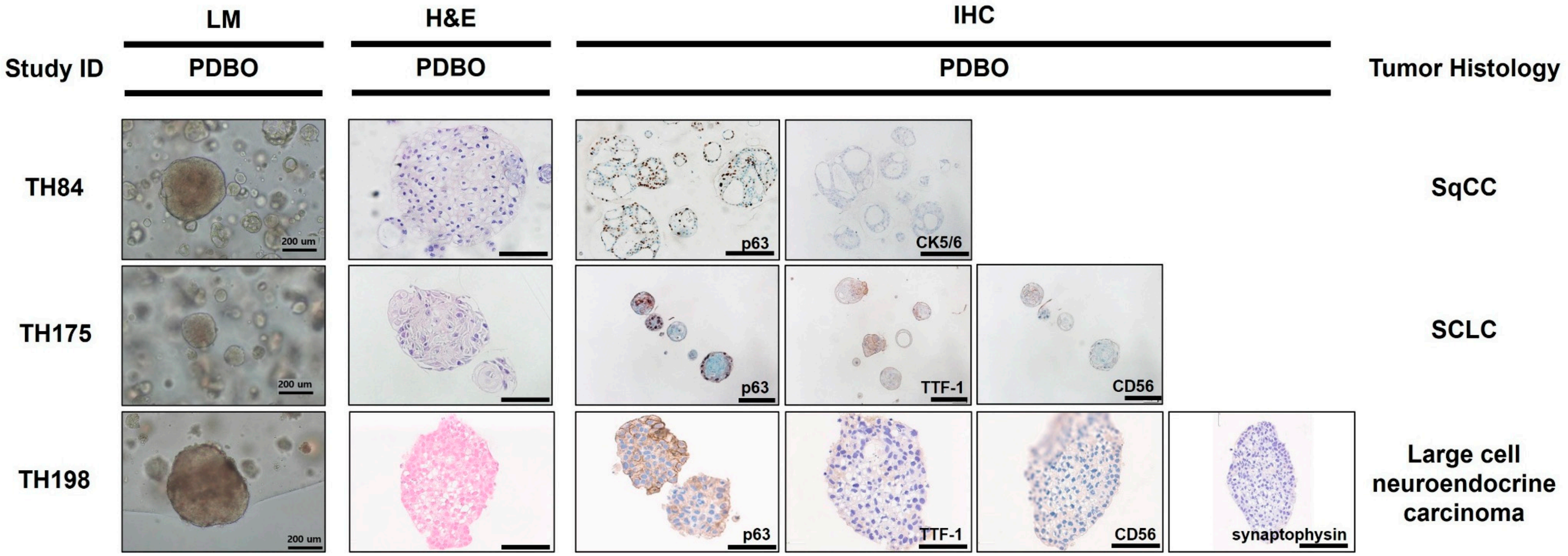

Figure S2. The representative results for LM (scale bar, 200 μm), H&E (magnification, x200), and IHC (scale bar, 200 μm) staining of PDBOs compared with H&E staining of patients' tumors. LM, light microscopy; H&E, hematoxylin and eosin; IHC, immunohistochemistry; PDBOs, patient-derived benign organoids; SqCC, squamous cell carcinoma; SCLC, small cell lung cancer.

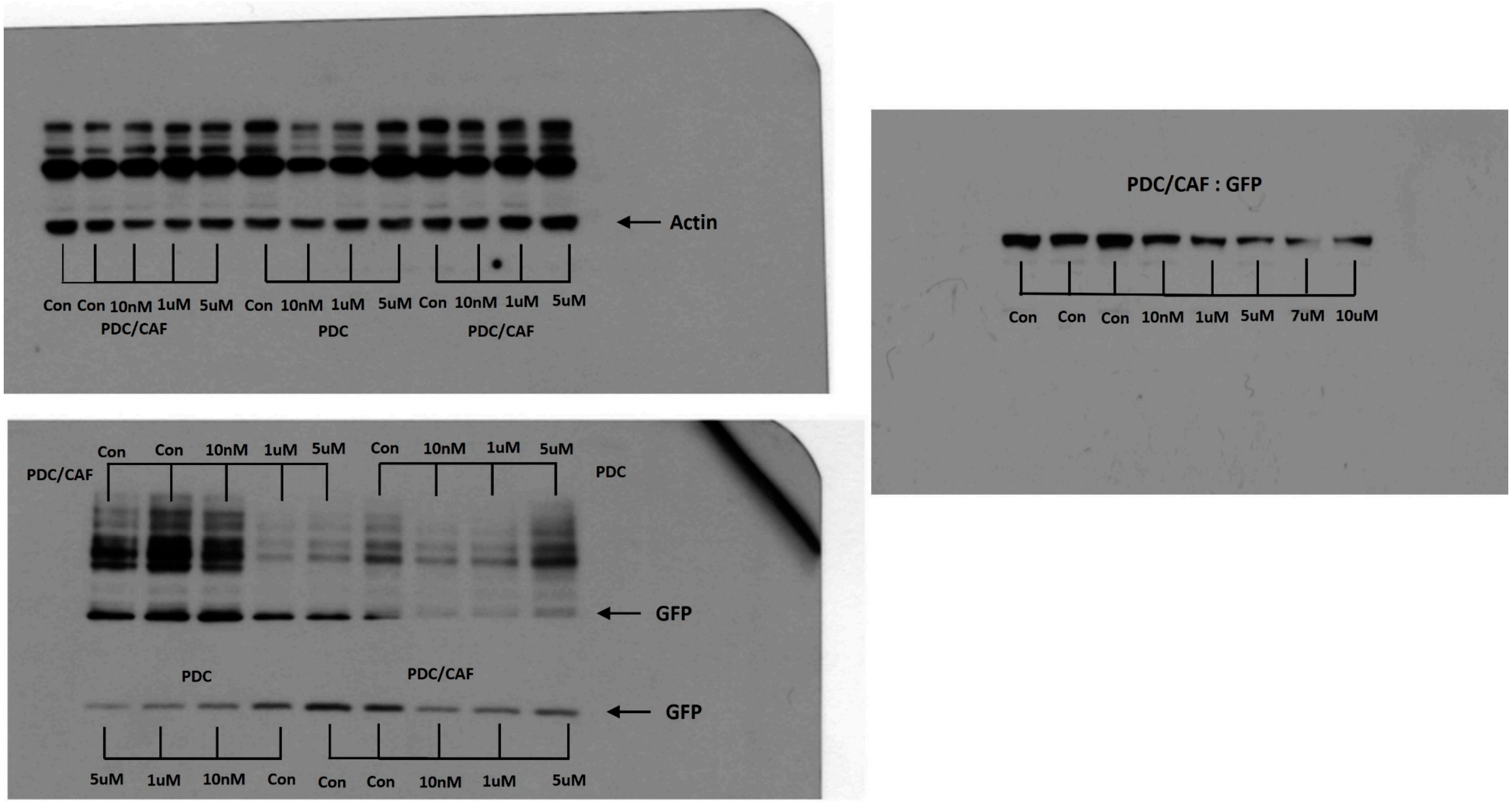

Figure S3. The original western blot image for Figure 4B.

PDTOs were cultured alone or co-cultured with CAFs and treated with increasing concentrations of paclitaxel (0, 10 nM, 1 μM, 5 μM). The untreated group (0 μM) was used as the control (Con).

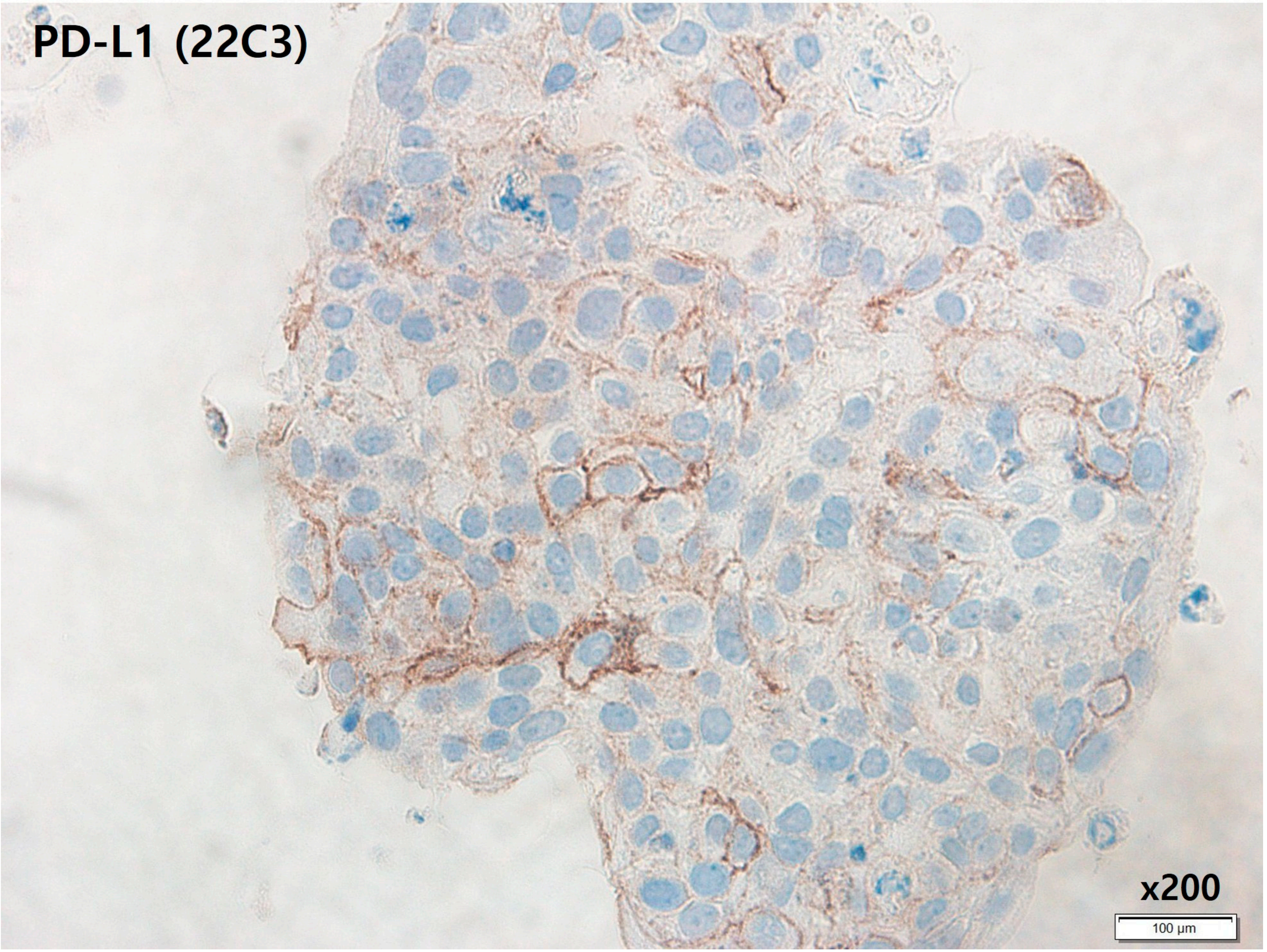

Figure S4. Representative IHC staining results of PDTOs (magnification, x200). PD-L1 (22C3), Dako Agilent

Table S1. Characteristics of successfully established PDTOs (N=25).

No.	Study ID	Histology	Lesion Location	Tissue acquisition method	Patient tumor genotype	PDTO genotype	PDTO IHC results
1	TH 64	ADC	4L LN	EBUS-TBNA	ALK V198A	ALK V198A	CK7+, TTF1+
2	TH 81	ADC	7 LN	EBUS-TBNA	KRAS G12C	KRAS G12C	CK7+, TTF1+
3	TH 89	ADC	Primary lesion	Surgery	ERBB2 R647K	ERBB2 R647K	TTF1+, CK7+
4	TH 101	ADC	Primary lesion	EBUS-TBNA	EGFR L858R	EGFR L858R	TTF1+, CK7+
5	TH 108	SCLC	Primary lesion	EBUS-TBNA	MET I284M	MET I284M	CD56+, Synaptophysin+
6	TH 122	SqCC	Primary lesion	Surgery	NA	NOTCH1 E948Q	p63+, CK5/6+
7	TH 133	SCLC	Primary lesion	EBUS-TBNA	ATM I1688T/ASXL1 E518V	ATM I1688T/ASXL1 E518V	CD56+, Synaptophysin+
8	TH 137	ADC	4R LN	Surgery	MET exon 14 skipping mutation	MET exon 14 skipping mutation	TTF1-, CK7+
9	TH 141	ADC	Primary lesion	Surgery	NA	SMARCA4 Y1472X	TTF1+, CK7+
10	TH 158	ADC	4R LN	EBUS-TBNA	TP53 Y236X, KIT N566K	TP53 Y236X, KIT N566K	TTF1-, CK7+
11	TH 167	ADC	Primary lesion	EBUS-TBNA	ERBB2 G776delinsVC	ERBB2 G776delinsVC	CD56+, Synaptophysin+

12	TH 174	ADC	Primary lesion	EBUS-TBNA	ROS1 fusion+	EZR-ROS fusion	TTF1+, CK7+
13	TH 176	SCLC	7 LN	Surgery	ALK M1478T	ALK M1478T	CD56-, Synaptophysin+
14	TH 189	SqCC	Primary lesion	Surgery	MSH6 T1355fs	MSH6 T1355fs	p63+, CK5/6+
15	TH 200	ADC	Primary lesion	EBUS-TBNA	EGFR exon 20 insertion (P772delinsPHV), ALK P666S	EGFR exon 20 insertion (P772delinsPHV), ALK P666S	TTF1+, CK7+
16	TH 221	SqCC	7 LN	Surgery	BRCA2 S3123G, MTOR Q1496L	BRCA2 S3123G, MTOR Q1496L	TTF1+, CK7+
17	TH 224	Mucinous ADC	Primary lesion	Surgery	KRAS G12D, SMAD4 528-529del	KRAS G12D, SMAD4 528-529del	TTF1+, CK7+
18	TH 235	SCLC	Primary lesion	EBUS-TBNA	BRAC2 E3377D	BRAC2 E3377D	CK5/6+, Synaptophysin+
19	TH 241	ADC	4R LN	EBUS-TBNA	ALK IHC 3+	ALK FISH (+)	TTF1+, CK7+
20	TH 269	SqCC	Primary lesion	Surgery	ATM N1680S	ATM N1680S	p63+, CK5/6+
21	TH 275	SqCC	Primary lesion	Surgery	NA	ERBB2 R217C	p63+, CK5/6+
22	TH 276	ADC	4R LN	EBUS-TBNA	NA	ATM R3008H NF1 Q400* ERBB4 A1213S POLE H708D	TTF1+, CK7+
23	TH 277	ADC	Primary lesion	Surgery	EGFR L858R	EGFR L858R	TTF1+, CK7+

24	TH 278	ADC	4R LN	EBUS-TBNA	NA	KRAS G12C<br>BARD1<br>359_365del<br>CDH1 L630V	TTF1+, CK7+
25	TH 285	ADC	Primary lesion	Surgery	EGFR exon 19 del	EGFR exon 19 del	TTF1+, CK7+

PDTOs, patient derived tumor organoids; ADC, adenocarcinoma; SqCC, squamous cell carcinoma; SCLC, small cell lung cancer; EBUS-TBNA, endobronchial ultrasound-guided transbronchial needle aspiration; NA, not applicable; L, left; R, right.

Table S2.

Targeted exome sequencing for tumor tissues and organoids

TH64

SNV/INDEL

Gene	Amino Acid Change	VAF, %		Class
		Tissue	Organoid	
ALK	V198A	31.51	28.91	Class 2
ATM	G3051*	25.81	26.18	Class 3, 4, 5
BRCA2	A2951T	56.72	60.68	Class 3, 4, 5
ASXL1	G642fs	5.81		Class 4
TET2	I274fs	4.56		Class 4
IDH2	R140Q	2.41		Class 4, 5
MET	A5T	56.75	65.7	Class 4, 5
APC	S559fs	31.04	47.6	Class 5
ABCB1	E275G		31.82	Novel
ATR	T2556S	44.04	41.16	Novel
AXIN2	761_765del	0.58		Novel
BLM	G512fs		0.83	Novel
BRD7	277_277del	2.42	2.19	Novel
DIS3	324_325del	12.14	33.78	Novel
EML4	S978L	44.89	63.18	Novel
EP300	Q935L		38.49	Novel
EPHB1	Q962L	28.78	48.79	Novel

CNV

Gene	Status		Class
	Tissue	Organoid	
KRAS		Amp.	Class 1, 2, 3, 4, 5
TYMS		Amp.	Class 2
SRSF2	Amp.	Amp.	Class 4
CDKN2A	Del.	Del.	Class 4, 5
PIK3CA		Amp.	Class 4, 5
CDKN2B	Del.	Del.	Class 5
BCL6		Amp.	Novel
CCND3		Amp.	Novel
CDKN1B		Amp.	Novel
CDKN2C		Amp.	Novel
ETV5		Amp.	Novel
FOXA1		Amp.	Novel
MAP3K13		Amp.	Novel
RET		Amp.	Novel
SOX2		Amp.	Novel
TFRC		Amp.	Novel

FGFR1	132_133del		0.83	Novel
FLT1	R73K	38.09	63.91	Novel
HDAC2	K11fs		0.71	Novel
INSR	Q61*	25.26		Novel
JAK2	D24Y	47.29		Novel
LATS1	P237Q	51.8	38.87	Novel
NCOR1	E546G	45.51		Novel
NTRK3	P719H	29.45	46.19	Novel
SETD2	N951D	41.18	32.18	Novel
SLC29A1	G441A		44.63	Novel
SLX4	1195_1195del	36.69	39.67	Novel
STAT4	splicing	1.54		Novel
TGFBR2	E125fs	3.21	3.45	Novel
TP53		50.24		Novel
XPC	34_35del	1.78		Novel

TH81

SNV/INDEL

Gene	Amino Acid Change	VAF, %		Class
		Tissue	Organoid	
KRAS	G12C	53.33	75.84	Class 1, 2, 3, 4, 5
RB1	R621S	43.81	45.31	Class 2
BRCA2	R18H	47.45	44.95	Class 3, 4, 5
BRCA2	G602fs		0.89	Class 3, 4, 5
AR	L57delinsLQ	13.75	10.53	Class 4
PTCH1	D898N	62.06	75.09	Class 4
ABL1	605_605del	1.21		Novel
ARID1B	Y1673C	40.61	34.46	Novel
BRD7	277_277del	2.7	2.7	Novel
DPYD	V335M	36.72		Novel
EGF	E1134fs	26.74		Novel
EP300	M755I	59.48		Novel
ERCC5	K915fs	1.1	0.96	Novel
FAT3	Y509C	42.52	29.13	Novel
JAK1	N226S	39.15		Novel
KDM5A	K1199fs	0.84		Novel
MSH3	A60delinsAAAP	35.65	62.83	Novel
NCOA3	1243_1244del	10.93	9.11	Novel
RAD50	L642V	66.95		Novel
RICTOR	R910H	53.06	65.57	Novel
SPEN	A2315V		27.37	Novel
STK11		31.36		Novel

CNV

Gene	Status		Class
	Tissue	Organoid	
SRSF2	Amp.		Class 4
KRAS	Amp.	Amp.	Class 1, 2, 3, 4, 5
NOTCH1		Amp.	Class 3, 4, 5
PTCH1		Amp.	Class 4
SMARCA4		Del.	Class 4
SRSF2		Amp.	Class 4
CDKN2A		Del.	Class 4, 5
GNAQ		Amp.	Class 4, 5
CDKN2B		Del.	Class 5
PTPRD		Del.	Class 5
RIT1		Amp.	Class 5
APLNR		Amp.	Novel
AREG		Del.	Novel
CDX2		Amp.	Novel
DPYD		Del.	Novel
ERCC6		Del.	Novel
ETS1		Amp.	Novel
FLI1		Amp.	Novel
FOXA1		Amp.	Novel
HNF1A		Amp.	Novel
IRS2		Amp.	Novel
JAK1		Del.	Novel

TGFBR2	E125fs	3.84	3.53	Novel

KCNJ5		Amp.	Novel
MCL1		Amp.	Novel
MLLT3		Del.	Novel
NKX2-1		Amp.	Novel
RET		Del.	Novel
RHBDF2		Amp.	Novel
RNF43		Amp.	Novel
TBX3		Amp.	Novel

TH89

SNV/INDEL

Gene	Amino Acid Change	VAF, %		Class
		Tissue	Organoid	
ERBB2	R647K	69.07	47.01	Class 2, 4, 5
CDKN2A	H66R	45.36	45.99	Class 4
FGFR3	T450M	49.19	50.24	Class 4
PTCH1	R893H	45.07	45.83	Class 4
BRAF	R384K	47.52	48.11	Class 4, 5
BARD1	359_365del	38.49	39.14	Class 5
CTNNB1	S33P	41		Novel
BRD7	277_277del	2.44	1.95	Novel
CIC	T1408A	50	40.97	Novel
CREBBP	T1688A	40.15	41.43	Novel
DICER1	G1526fs	28.85		Novel
DICER1	D1810Y	38.15		Novel
DPYD	V732I	46.31	43.55	Novel
EGF	E1134fs	29.79	29.4	Novel
ERBB3	A304S	47.35	47.82	Novel
FAT1	I2531V	46.76	49.1	Novel
GEN1	839_840del	42.42	43.89	Novel
IGF2R	S1194L	46.15	44.87	Novel
IL7R	S105N	43.99	39.19	Novel
KMT2A	G3131S	48.18	48.13	Novel
NTRK1	M566T	43.09	41.97	Novel

PLCG1	Y518H	46.97	49.74	Novel
SETD2	R400Q	45.8	45.67	Novel
ZBTB16	L153V	45.86	46.59	Novel
MSH3	K381fs		0.84	Novel

TH101

SNV/INDEL

Gene	FUNCTION	Amino Acid Change	VAF, %		Class
			Tissue	Organoid	
EGFR	nonsynonymous SNV	L858R	11.68	54.46	Class 1, 2, 4, 5
EGFR	nonsynonymous SNV	G863D		54.45	Class 2, 4, 5
EGFR	nonsynonymous SNV	K1179E	46.19	56.34	Class 2, 4, 5
PBRM1	nonsynonymous SNV	K1257N	46.54	47.61	Class 4
BCL2A1	frameshift insertion	L17fs	41.73	38.92	Novel
BRD7	frameshift deletion	277_277del	2.75	3.71	Novel
FANCA	nonsynonymous SNV	H305Y	45.5	40.48	Novel
FAT3	frameshift deletion	A1550fs	3.54	29.63	Novel
FAT3	nonsynonymous SNV	R3674W	47.41	50.9	Novel
GEN1	frameshift deletion	839_840del	41.17		Novel
KAT6A	nonframeshift deletion	1109_1110del	46.78	49.51	Novel
KDM5A	nonsynonymous SNV	P1474T	47.82	48.34	Novel
MSH3	frameshift deletion	K381fs	0.47		Novel
LRP1B	nonsynonymous SNV	R438S		28.76	Novel
NOTCH4	nonsynonymous SNV	S415F	47.06	48.48	Novel
POLD1	nonsynonymous SNV	A215V	44.58	42.45	Novel
SDHD	nonsynonymous SNV	L85F	49.29	44.12	Novel
SETBP1	nonsynonymous SNV	R627C	45.08	29.41	Novel
SETBP1	nonsynonymous SNV	S1337T	45.33	29.39	Novel
STAT4	NA	splicing	1.84		Novel
TGFBR2	frameshift deletion	E125fs	3.14	4.4	Novel

CNV

Gene	Status		Class
	Tissue	Organoid	
CDK4		Amp.	Class 4
CDKN2A		Del.	Class 4, 5
TOP1		Amp.	Class 4, 5
CDKN2B		Del.	Class 5
MDM2	Amp.	Amp.	Class 5
AREG	Del.	Del.	Novel
FCGR2B	Del.		Novel
CARD11		Amp.	Novel
ETV1		Amp.	Novel
FRS2	Amp.	Amp.	Novel
NKX2-1		Amp.	Novel
PLCG1		Amp.	Novel
RAC1		Amp.	Novel

XPA	nonsynonymous SNV	E106K	48.84	46.55	Novel

TH108

SNV/INDEL

Gene	Amino Acid Change	VAF, %		Class
		Tissue	Organoid	
TP53	R273H	95.05		Class 2, 3, 4, 5
MET	I284M	62.71	62.47	Class 4, 5
CCND3	269_271del	18.34	30.3	Class 5
ABL2	P981R	43.8	47.71	Novel
BLM	A915V	41.9	48.46	Novel
BRD7	277_277del	2.3	2.55	Novel
DICER1	E422K	46.04	47.25	Novel
ERCC2	V231M	54.88	66.85	Novel
KMT2D	P443Q	52.23	58.5	Novel
LRP1B	R3361Q	32.93	31.93	Novel
MSH2	Q419K	36.26	32.46	Novel
MSH3	A60delinsAAAP	36.89		Novel
NCOA3	1243_1246del	31.88	47.79	Novel
POLQ	G2033V	29.84	34.08	Novel
TGFBR2	E125fs	2.54	4.44	Novel

CNV

Gene	Status		Class
	Tissue	Organoid	
CEBPA	Amp.	Amp.	Class 4
CCNE1	Amp.	Amp.	Class 4, 5
PIK3CA	Amp.		Class 4, 5
AKT2	Amp.	Amp.	Class 5
ATR	Amp.	Amp.	Novel
BCL6	Amp.	Amp.	Novel
CBLB	Amp.	Amp.	Novel
CUL4A	Amp.	Amp.	Novel
EPHA3	Amp.	Amp.	Novel
EPHA6	Amp.	Amp.	Novel
EPHB1	Amp.	Amp.	Novel
ETV5	Amp.		Novel
FOXL2	Amp.	Amp.	Novel
GATA2	Amp.	Amp.	Novel
IRS2	Amp.	Amp.	Novel
LAMP1	Amp.	Amp.	Novel
MAP3K13	Amp.	Amp.	Novel
PIK3CB	Amp.	Amp.	Novel
POLQ	Amp.		Novel
SOX2	Amp.		Novel
TFRC	Amp.	Amp.	Novel

AREG		Del.	Novel

TH122

SNV/INDEL

Gene	Amino Acid Change	VAF, %		Class
		Tissue	Organoid	
TP53	R158L	28.41		Class 2, 3, 4, 5
ASXL1	G643fs	1		Class 4
NOTCH1	E948Q		52.03	Class 3, 4
FGFR3	T450M	44.71	96.31	Class 4
TET2	E1151*	37.7	47.09	Class 4
GNAQ	M59L		0.95	Class 4, 5
ABCB1	G960R		27.96	Novel
ABCC1	splicing	0.44		Novel
ABL1	605_605del	0.81	1.12	Novel
ASNS	V124L		0.98	Novel
AURKB	R44H	28.03		Novel
BRD7	277_277del	3.85	2.8	Novel
CBFB	E163G	47.04	30.14	Novel
CHD4	119_119del	0.64		Novel
EGF	W614L		43.23	Novel
EGF	E1134fs	31.57	31.64	Novel
FAT1	S2353A	47.52	47.36	Novel
FGFR1	132_133del	0.4		Novel
HDAC2	K11fs	0.52		Novel
IGF2R	A2459V	39.69		Novel

CNV

Gene	Status		Class
	Tissue (FFPE)	Organoid	
CCND1		Amp.	Class 2, 4, 5
FGF3		Amp.	Class 4
TTF1	Amp.		Class 2
PMS2	Amp.		Novel
RAC1	Amp.		Novel
ATR		Amp.	Novel
FGF19		Amp.	Novel
FGF4		Amp.	Novel
FOXL2		Amp.	Novel
GATA2		Amp.	Novel
MAP3K13		Amp.	Novel
SOX2		Amp.	Novel
TNKS		Del.	Novel

IGF2R	T1314fs	0.43		Novel
KDM5A	K1199fs	0.53	0.56	Novel
LRP1B	N1111K		52.4	Novel
LRP1B	C1103F		44.97	Novel
MDC1	P1100S	37.98		Novel
MSH2	T668I	43.54	63.43	Novel
MSH3	A60delinsAAAP	23.36	60.95	Novel
NTRK1	R593W	37.34	36.63	Novel
POLD1	V861M	48.49		Novel
RICTOR	P1668L	44.02	46.22	Novel
SDHC	V103L	0.94	0.85	Novel
SLX4	Q377H	0.85		Novel
TGFBR2	E125fs	4.48	4.11	Novel
TP53BP1	D34N	32.58		Novel
WRN	L628V	39.49		Novel
XPC	34_35del	1.62		Novel
ZNRF3	450_451del	1.01		Novel
ZNF217	Q300P		32.92	Novel

TH133

SNV/INDEL

Gene	FUNCTION	Amino Acid Change	VAF, %		Class
			Tissue	Organoid	
ATM	nonsynonymous SNV	I1688T		24.44	Class 3, 4, 5
ASXL1	nonsynonymous SNV	E518V	33.66	31.05	Class 4
ABCC3	nonsynonymous SNV	R1299Q	48.3	48.1	Novel
BRD7	frameshift deletion	277_277del	2.49	5.1	Novel
CDKN1B	nonsynonymous SNV	A156V	35	30.92	Novel
DDR2	nonsynonymous SNV	Q573L	25.7	29.85	Novel
EPHA2	nonsynonymous SNV	R566H	35.71	39.13	Novel
EPHB6	nonframeshift insertion	S173delinsSSS	25.32	22.76	Novel
EPHB6	nonframeshift insertion	P165delinsPSS	37.8	35.13	Novel
ERCC5	frameshift deletion	K915fs	0.5		Novel
ERCC5	frameshift insertion	V914fs	0.43		Novel
FANCM	nonsynonymous SNV	R581C	71.48	78.67	Novel
FAT1	nonsynonymous SNV	V3245A	70.01	59.64	Novel
FAT1	nonsynonymous SNV	S1803*	63.83	66.6	Novel
FAT1	stopgain SNV	D1113N	67.99	65.15	Novel
FAT1	stopgain SNV	Q4114*		29.08	Novel
GSTP1	nonsynonymous SNV	D147Y	69.76	71	Novel
IL7R	nonsynonymous SNV	F213S	33.5	30.56	Novel
ITK	frameshift deletion	S58fs	44.89	47.46	Novel
JAK1	nonsynonymous SNV	N973K	30.55	33.49	Novel
NCOA3	nonframeshift deletion	1243_1244del	22.37	17	Novel

CNV

Gene	Status		Class
	Tissue	Organoid	
TERT	Amp.	Amp.	Class 4
RICTOR		Amp.	Class 5
ATR		Amp.	Novel
BCL6		Amp.	Novel
CBLB		Amp.	Novel
CDKN2B		Amp.	Novel
CUL4A	Amp.	Amp.	Novel
DIS3	Amp.	Amp.	Novel
EPHB1		Amp.	Novel
ERCC5	Amp.	Amp.	Novel
FGF10	Amp.	Amp.	Novel
FGF14	Amp.	Amp.	Novel
FOXA1	Amp.	Amp.	Novel
FOXL2		Amp.	Novel
GATA2		Amp.	Novel
HIF1A		Amp.	Novel
HSP90AA1		Amp.	Novel
IL7R		Amp.	Novel
IRS2	Amp.	Amp.	Novel
LAMP1	Amp.	Amp.	Novel
MAP3K13		Amp.	Novel

RB1	splicing		95.31		Novel
ROS1	nonsynonymous SNV	T326R	59.22	57.92	Novel
SETD2	nonsynonymous SNV	G121A	44.03	47.08	Novel
TGFBR2	frameshift insertion	E125fs	0.95		Novel
TGFBR2	frameshift deletion	E125fs	3.31	3.1	Novel
TOP2A	nonsynonymous SNV	R635H	48.14	48.11	Novel
TP53	splicing		93.2		Novel
TP53BP1	nonsynonymous SNV	M716V	49.71	52.73	Novel

PIK3CB		Amp.	Novel
TFRC		Amp.	Novel

TH137

SNV/INDEL

Gene	FUNCTION	Amino Acid Change	VAF, %		Class
			Tissue	Organoid	
TP53	nonsynonymous SNV	R282W	32.96		Class 2, 3, 4, 5
MET	frameshift deletion	1010_1010del	67.25	99.9	Class 2, 4, 5
ATM	nonsynonymous SNV	K2749I	48.35	40.19	Class 3, 4, 5
SMAD4	nonsynonymous SNV	I40V	37.19		Class 4
BCL2L2	nonsynonymous SNV	T175M	55.83	50.96	Novel
BLM	frameshift deletion	G512fs	1.12		Novel
BRD7	frameshift deletion	277_277del	1.65	6.5	Novel
CCND3	nonsynonymous SNV	I208T	46.62	42.95	Novel
DICER1	nonsynonymous SNV	H145Y	50.11	48.68	Novel
ERCC6	nonsynonymous SNV	R837H	69.81		Novel
ESR1	nonsynonymous SNV	P146Q	48.7	52.94	Novel
ETV6	nonsynonymous SNV	R191L	44.43	35	Novel
FANCM	nonsynonymous SNV	Q1175H	49.39	53.5	Novel
GEN1	nonsynonymous SNV	G325V	45.79	46.92	Novel
GEN1	frameshift deletion	839_840del	43.21	39.52	Novel
HSP90AA1	nonframeshift deletion	241_241del	37.79	41.44	Novel
IGF2R	nonsynonymous SNV	A615V	48.19	48.66	Novel
LTK	nonsynonymous SNV	R569H	32.78	33.1	Novel
MDC1	nonsynonymous SNV	R1079C	49.46	46.01	Novel
PPP1R15A	nonsynonymous SNV	R594C	71.95		Novel
TGFBR2	nonframeshift insertion	E567delinsEH	55.34	69.67	Novel

CNV

Gene	Status		Class
	Tissue	Organoid	
EGFR		Amp.	Class 2, 4, 5
SMAD4		Del.	Class 4
TERT	Amp.	Amp.	Class 4
CDKN2A		Del.	Class 4, 5
MET		Amp.	Class 4, 5
ASNS		Amp.	Class 5
CDKN2B		Del.	Class 5
RICTOR		Amp.	Class 5
AREG		Del.	Novel
CARD11		Amp.	Novel
CBFB		Del.	Novel
CDK6		Amp.	Novel
EPHB4		Amp.	Novel
ETV1	Amp.	Amp.	Novel
FGF10		Amp.	Novel
HGF		Amp.	Novel
IKZF1		Amp.	Novel
IL7R		Amp.	Novel
PIK3CG		Amp.	Novel
PMS2		Amp.	Novel
RAC1		Amp.	Novel

TH141

SNV/INDEL

Gene	FUNCTION	Amino Acid Change	VAF, % (Organoid)	Class
SMARCA4	stopgain SNV	Y1472X	47.6	Class 2, 4
SF3B1	nonsynonymous SNV	L743F	40.43	Class 3, 4
POLE	nonsynonymous SNV	S711F	38.31	Class 5
PTPRD	nonsynonymous SNV	G1418W	45.87	Class 5
ABCC1	nonsynonymous SNV	F191V	45.58	Novel
AKT1	nonsynonymous SNV	Q454E	62.47	Novel
BACH1	nonsynonymous SNV	P268L	43.31	Novel
BRD7	frameshift deletion	277_277del	3.26	Novel
CBL	nonsynonymous SNV	S794F	66.22	Novel
DICER1	nonsynonymous SNV	A1185T	30.85	Novel
EPHA7	nonsynonymous SNV	Y791C	31.87	Novel
FGF23	stopgain SNV	Q208*	50.35	Novel
FGFR1	nonframeshift deletion	165_166del	0.6	Novel
HSP90AA1	nonframeshift deletion	279_280del	0.96	Novel
IGF2R	frameshift deletion	F1120fs	20.66	Novel
KMT2A	nonsynonymous SNV	G73E	60	Novel
KMT2D	nonsynonymous SNV	E1352K	30.65	Novel
MSH2	nonsynonymous SNV	P5Q	71.11	Novel
MSH3	frameshift deletion	K381fs	0.47	Novel
NCOA3	nonframeshift deletion	1243_1244del	1.21	Novel
NCOR1	splicing		48.6	Novel
NOTCH3	frameshift deletion	R107fs	29.73	Novel

CNV

Gene	Status Organoid	Class
	TISSUE	
TYMS	Amp.	Class 2
BCL2	Del.	Class 4
ABCB1	Amp.	Novel
CCND3	Amp.	Novel
CDK6	Amp.	Novel
FANCE	Amp.	Novel
NBN	Amp.	Novel
SETBP1	Del.	Novel

XRCC3	nonsynonymous SNV	E26G	61.63	Novel

TH158

SNV/INDEL

No.	Gene	Amino Acid Change	FUNCTION	VAF, %		Class
				Tissue	Organoid	
1	TP53	Y236X	stopgain SNV	35.85	46.95	Class 2, 3, 4, 5
2	KIT	N566K	nonsynonymous SNV	0.85	0.6	Class 2, 4, 5
3	ATM	E2272K	nonsynonymous SNV	0.82	0.83	Class 3, 4, 5
4	TET2	M1789I	nonsynonymous SNV		0.95	Class 4
5	STK11	P281L	nonsynonymous SNV	82.15		Class 4
6	MET	C95S	nonsynonymous SNV	0.68		Class 4, 5
7	ABCG2	E60G	nonsynonymous SNV		0.69	Novel
8	ARID2	H641N	nonsynonymous SNV		0.7	Novel
9	ATR	Q1377L	nonsynonymous SNV		0.62	Novel
10	BRD7	277_277del	frameshift deletion	2.65	2.62	Novel
11	CBL	P548L	nonsynonymous SNV	41.98	54.61	Novel
12	CDH2	E278*	stopgain SNV		0.82	Novel

CNV

Gene	Status		Class
	Tissue	Organoid	
ATM	Amp.	Amp.	Class 3, 4, 5
BCL2		Del.	Class 4
TERT		Amp.	Class 4
CDKN2A	Del.	Del.	Class 4, 5
APC		Amp.	Class 5
CDH1		Amp.	Class 5
CDKN2B	Del.	Del.	Class 5
PTPRD	Del.	Del.	Class 5
AXIN1		Amp.	Novel
CCND3		Amp.	Novel
ETV6		Del.	Novel
IDO1	Amp.	Amp.	Novel

13	DDR2	S841*	stopgain SNV	0.44		Novel
14	DDR2	Q816K	nonsynonymous SNV		0.51	Novel
15	EGF	C745Y	nonsynonymous SNV		0.74	Novel
16	EGF	E1134fs	frameshift insertion	38.43	41.38	Novel
17	EPHA3	S419R	nonsynonymous SNV	62.32		Novel
18	ERCC4	R86S	nonsynonymous SNV	0.83		Novel
19	ERCC4	E91K	nonsynonymous SNV		27.99	Novel
20	ERCC6	A1058T	nonsynonymous SNV		0.72	Novel
21	FRS2	S155*	stopgain SNV		0.66	Novel
22	HSP90AA1	E537*	stopgain SNV		0.64	Novel
23	GSTP1	D147Y	nonsynonymous SNV	37.97		Novel
24	IDO1	S315*	stopgain SNV	0.55	0.68	Novel
25	IL7R	W264G	nonsynonymous SNV	0.7	0.54	Novel
26	INPP4B	A875E	nonsynonymous SNV	58.1	75.36	Novel
27	KDM5A	P1474T	nonsynonymous SNV	70.42		Novel

IL7R		Amp.	Novel
KAT6A	Amp.	Amp.	Novel
MUTYH	Amp.	Amp.	Novel
NBN		Amp.	Novel
NRG1	Amp.	Amp.	Novel
RAD21		Amp.	Novel
RET	Amp.	Amp.	Novel
SERPINB3		Del.	Novel
SOCS1		Amp.	Novel

28	KMT2A	S1271N	nonsynonymous SNV	0.81		Novel
29	KMT2A	E1268*	stopgain SNV		0.81	Novel
30	KMT2A	E1269G	nonsynonymous SNV		0.84	Novel
31	LRP1B	A337V	nonsynonymous SNV		0.92	Novel
32	LRP1B	E3379K	nonsynonymous SNV		0.72	Novel
33	LATS1	C686S	nonsynonymous SNV	0.67		Novel
34	LTK	A654T	nonsynonymous SNV	80.1		Novel
35	MDC1	E952*	stopgain SNV		0.72	Novel
36	MDC1	T1891A	nonsynonymous SNV	47.28	42.53	Novel
37	MSH3	K381fs	frameshift deletion		0.52	Novel
38	MYCN	P358L	nonsynonymous SNV	56.18	45.68	Novel
39	NSD1	C1725F	nonsynonymous SNV		0.94	Novel
40	NOTCH2	C1472Y	nonsynonymous SNV	0.66		Novel
41	NOTCH3	D221Y	nonsynonymous SNV	59.69		Novel
42	NSD1	Q2579E	nonsynonymous SNV	85.44		Novel

43	NTRK3	N392I	nonsynonymous SNV	62.54		Novel
44	PDGFRA	K194*	stopgain SNV	0.8		Novel
45	PKHD1	R2052*	stopgain SNV		27.89	Novel
46	PKHD1	G2648S	nonsynonymous SNV	48.08	51.73	Novel
47	PKHD1	S3018Y	nonsynonymous SNV		0.59	Novel
48	POLQ	Q1876*	stopgain SNV	0.96	0.98	Novel
49	SDHC	H114L	nonsynonymous SNV	0.75	0.58	Novel
50	SETBP1	R531M	nonsynonymous SNV	62.41		Novel
51	SOX2	P302T	nonsynonymous SNV	74.14		Novel
52	SPEN	R653L	nonsynonymous SNV	0.93		Novel
53	SPEN	R655K	nonsynonymous SNV	0.94	0.76	Novel
54	STAT4	R113G	nonsynonymous SNV	47.9	46.15	Novel
55	STAT4	splicing	NA	1.16		Novel
56	UGT1A1	Y486D	nonsynonymous SNV	39.67	46.76	Novel

TH167

SNV/INDEL

Gene	FUNCTION	Amino Acid Change	VAF, %		Class
			Tissue	Organoid	
ERBB2	nonframeshift insertion	G776delinsVC	87.5	84.66	Class 2, 4, 5
BRCA2	nonsynonymous SNV	V208G	46.36	47.5	Class 3, 4, 5
FGFR3	nonsynonymous SNV	L164V	42.54	50.83	Class 4
ABCC1	nonsynonymous SNV	Y490H	28.65	30.32	Novel
BRD7	frameshift deletion	277_277del	3.17	4.03	Novel
EGF	frameshift insertion	E1134fs	9.15	7.58	Novel
EPHB6	nonframeshift insertion	S173delinsSSS	18.66	17.53	Novel
EPHB6	nonframeshift insertion	P165delinsPSS	30.4	28.53	Novel
HDAC2	frameshift deletion	K11fs		1.29	Novel
KAT6A	nonframeshift deletion	1109_1110del	6.88	8.28	Novel
MSH3	nonframeshift insertion	A60delinsAAAP	22.11	17.41	Novel
NCOA3	nonframeshift deletion	1243_1244del	24.36		Novel
NCOR1	nonsynonymous SNV	Q26K	0.96		Novel
NRG1	nonsynonymous SNV	R56Q	92.86	86.46	Novel
PIK3CB	nonsynonymous SNV	F979Y	41.52	39.06	Novel
PML	nonsynonymous SNV	V345M	62.3	61.64	Novel
RICTOR	nonsynonymous SNV	R855Q	46.87	44.36	Novel
TP53BP1	nonsynonymous SNV	P1059L	61.09	57.69	Novel
TTF1	nonsynonymous SNV	M429K	59.26	52.75	Novel
TTF1	nonsynonymous SNV	M429I	59.23	52.17	Novel
TTF1	nonsynonymous SNV	E430K	59.57	52.68	Novel

CNV

Gene	Status		Class
	Tissue	Organoid	
TYMS	Amp.	Amp.	Class 2
NTRK1	Amp.	Amp.	Class 4
CCNE1	Amp.	Amp.	Class 4, 5
ABCC3	Amp.	Amp.	Class 5
AKT2	Amp.	Amp.	Class 5
RIT1		Amp.	Class 5
APCDD1	Amp.	Amp.	Novel
CDK12		Amp.	Novel
CD274	Del.		Novel
DDR2	Amp.	Amp.	Novel
JAK2	Del.	Del.	Novel
MLLT3	Amp.	Amp.	Novel
PDCD1LG2	Del.	Del.	Novel
SPOP	Amp.	Amp.	Novel
ZNF217	Amp.	Amp.	Novel

XPC	nonframeshift deletion	34_35del		1.92	Novel

TH174

SNV/INDEL

Gene	FUNCTION	Amino Acid Change	VAF, %		Class
			Tissue	Organoid	
TP53	stopgain SNV	E224*		74.88	Class 2, 3, 4, 5
TP53	frameshift insertion	V218fs		12.44	Class 2, 3, 4, 5
ASXL1	nonsynonymous SNV	C1204W		63.32	Class 4
APC	nonsynonymous SNV	L2384I		48.17	Class 5
ABL2	nonsynonymous SNV	P996R		41.3	Novel
BRD7	frameshift deletion	277_277del		4.46	Novel
CSF1R	frameshift insertion	Q970fs		41.72	Novel
EPHB6	nonsynonymous SNV	P349S		67.91	Novel
FANCM	nonsynonymous SNV	I917V		46.81	Novel
GEN1	frameshift deletion	839_840del		29.5	Novel
MTHFR	nonsynonymous SNV	R473W		44.57	Novel
MUS81	nonsynonymous SNV	V388I		47.41	Novel
PIK3R2	nonsynonymous SNV	P151S		47.13	Novel
RAD54L	nonsynonymous SNV	K12R		41.22	Novel
SLX4	nonsynonymous SNV	A1726V		30.97	Novel
TTF1	nonsynonymous SNV	A246V		45.03	Novel

CNV

Gene	Status		Class
	Tissue	Organoid	
CDKN2A		Del.	Class 4, 5
CDKN2B		Del.	Class 5
APLNR		Amp.	Novel
IGF2		Amp.	Novel

Fusion

Gene		Class
Tissue	Organoid	
	EZR > ROS1	Novel

TH176

SNV/INDEL

Gene	FUNCTION	Amino Acid Change	VAF, %		Class
			Tissue	Organoid	
ALK	nonsynonymous SNV	D602E	25.35		Class 2
ALK	nonsynonymous SNV	M1478T	43.81	48.66	Class 2
RB1	stopgain SNV	R251*	61.26		Class 2
BRCA1	nonsynonymous SNV	Y856H	79.81	48.4	Class 3, 4
ASXL1	nonsynonymous SNV	P913A	41.57	44.7	Class 4
BARD1	nonsynonymous SNV	M621T	43.73	49.15	Novel
BRD7	frameshift deletion	277_277del	2.23	2.87	Novel
CDKN2B	nonsynonymous SNV	D138G	55.6	47.23	Novel
CSF1R	nonsynonymous SNV	G936S		40.22	Novel
ESR1	nonsynonymous SNV	P146Q	48.82	50.91	Novel
IRS2	nonsynonymous SNV	P813S		53.9	Novel
FANCI	nonsynonymous SNV	G346V	27.38		Novel
IRF1	nonsynonymous SNV	N49I	61.08		Novel
KMT2D	nonsynonymous SNV	T245S	44.97	43.43	Novel
LRP1B	nonsynonymous SNV	V3868M	39.45	48.05	Novel
LRP1B	nonsynonymous SNV	I761V	50.08	43.25	Novel
MSH3	nonframeshift deletion	55_57del	5.67	20.26	Novel
NCOA3	nonframeshift deletion	1243_1244del	21.55		Novel
NCOA3	nonframeshift insertion	M1243delinsMQ	9.57	9.23	Novel
NTRK2	nonsynonymous SNV	A678E	24.83		Novel
PRKCB	nonsynonymous SNV	R624Q	47.12	47.65	Novel
RRAS2	nonsynonymous SNV	R117H		48.52	Novel

CNV

Gene	Status		Class
	Tissue	Organoid	
TERT	Amp.		Class 4
RICTOR	Amp.		Class 5
EPHA2	Amp.		Novel
FGF10	Amp.		Novel

SLC29A1	nonsynonymous SNV	A435D	26.87		Novel
TP53	splicing		63.41		Novel
TP53BP1	nonsynonymous SNV	I1179V	49.67	48.24	Novel
WEE1	nonsynonymous SNV	R481P	60.4		Novel

TH189

SNV/INDEL

Gene	FUNCTION	Amino Acid Change	VAF, %		Class
			Tissue	Organoid	
MSH6	frameshift insertion	T1355fs	50.48	44.63	Class 5
ABL1	nonsynonymous SNV	G706V	46.63	49.41	Novel
BLM	frameshift deletion	G512fs		1.04	Novel
BRD7	frameshift deletion	277_277del	4.33	2.82	Novel
BRIP1	nonsynonymous SNV	A144T	53.22	46.44	Novel
CDKN1B	nonsynonymous SNV	A167S	41.4	43.57	Novel
DNMT1	nonsynonymous SNV	G860R	45.92	51.13	Novel
EGF	frameshift insertion	E1134fs	47.36	47.45	Novel
EP300	nonsynonymous SNV	S507G	41.37	43.83	Novel
GATA3	nonsynonymous SNV	A395T	44.72	50.44	Novel
IGF2R	nonsynonymous SNV	A2459V	36.56	44.98	Novel
KAT6A	nonframeshift deletion	1109_1110del	48.72	44.71	Novel
KMT2A	nonsynonymous SNV	G3131S	40.75	46.65	Novel
NOTCH3	nonsynonymous SNV	R75Q	38.51	44.51	Novel
PKHD1	nonsynonymous SNV	R559W	46.39	44.87	Novel
SLX4	nonframeshift deletion	1195_1195del	39.63	45.07	Novel
SPEN	frameshift deletion	803_803del		1.18	Novel

CNV

Gene	Status		Class
	Tissue	Organoid	
EGFR	Amp.		Class 2, 4, 5

TH200

SNV/INDEL

Gene	FUNCTION	Amino Acid Change	VAF, %		Class
			Tissue	Organoid	
ALK	nonsynonymous SNV	P666S	60.07	50.66	Class 2
EGFR	nonframeshift insertion	P772delinsPHV	68.95	0.91	Class 2, 4, 5
MET	NA	del		0.14	Class 4, 5
BRD7	frameshift deletion	277_277del	3.77	3.93	Novel
CDH2	nonsynonymous SNV	N845S	61.12	46.87	Novel
IGF2R	nonsynonymous SNV	P1004A	48.83	49.4	Novel
IKBKE	nonsynonymous SNV	G660E	67.99	50.52	Novel
KAT6A	nonframeshift deletion	1109_1110del	47.93	44.17	Novel
MSH3	frameshift deletion	K381fs	0.68		Novel
NCOA3	stopgain SNV	Y1419_C1420delinsX	30.63	29.18	Novel
PARP3	nonframeshift deletion	231_233del	51.74	40.28	Novel
PDGFRB	nonsynonymous SNV	R685C	46.46	48.78	Novel
PKHD1	nonsynonymous SNV	I2364N	59.9	45.99	Novel
PKHD1	nonsynonymous SNV	S3210C	59.92	45.93	Novel
RHBDF2	nonsynonymous SNV	S292F	34.18	45.93	Novel
SLX4	nonsynonymous SNV	P929L	37.47	46.38	Novel

CNV

Gene	Status		Class
	Tissue	Organoid	
EGFR	Amp.		Class 2, 4, 5
MDM2	Amp.		Class 5
CARD11	Amp.		Novel
ETV1	Amp.		Novel
FANCM	Amp.		Novel
FOXA1	Amp.		Novel
FRS2	Amp.		Novel
HIF1A	Amp.		Novel
IKZF1	Amp.		Novel
MAD1L1	Amp.		Novel
MYC	Amp.		Novel
NBN	Amp.		Novel
PMS2	Amp.		Novel
RAC1	Amp.		Novel
RAD21	Amp.		Novel
TSHR	Amp.		Novel

TH221

SNV/INDEL

No.	Gene	Amino Acid Change	FUNCTION	VAF, %		Class
				Tissue	Organoid	
1	BRCA2	S3123G	nonsynonymous SNV	49.34	47.23	Class 3, 4, 5
2	MTOR	Q1496L	nonsynonymous SNV	48.54	48.5	Class 4, 5
3	ABL1	605_605del	nonframeshift deletion		1.07	Novel
4	APCDD1	S353L	nonsynonymous SNV	46.39	50.29	Novel
5	AXIN2	S738F	nonsynonymous SNV	45.4	49.93	Novel
6	BRD7	277_277del	frameshift deletion	2.77	2.14	Novel
7	EGF	E1134fs	frameshift insertion	30.64	30.88	Novel
8	ERCC3	S735P	nonsynonymous SNV	46.3	45.56	Novel
9	ERCC5	K915fs	frameshift deletion		0.99	Novel
10	FAT3	G1712E	nonsynonymous SNV	47.46	46.08	Novel
11	FAT3	R2058H	nonsynonymous SNV	49.28	49.05	Novel
12	FLT1	A673V	nonsynonymous SNV	45.11	44.56	Novel
13	GEN1	839_840del	frameshift deletion	44.28	45.72	Novel
14	KMT2D	R5392H	nonsynonymous SNV	48.1	46.02	Novel
15	LRP1B	L2349V	nonsynonymous SNV	45.74	48.15	Novel
16	MSH3	A60delinsAAAP	nonframeshift insertion		22.83	Novel
17	NCOA3	1243_1246del	nonframeshift deletion	31.41	25.84	Novel
18	PALB2	L763F	nonsynonymous SNV	46.12	48.76	Novel
19	PMS2	A127T	nonsynonymous SNV	43.52	47.45	Novel
20	RAD50	I118T	nonsynonymous SNV	44.24	47.83	Novel
21	SPEN	D1234N	nonsynonymous SNV	45.33	46.91	Novel

22	TP53BP1	N258H	nonsynonymous SNV	44.79	47.13	Novel
23	WRN	R857C	nonsynonymous SNV	45.17	47.33	Novel

TH224 P2

SNV/INDEL

No.	Gene	Amino Acid Change	FUNCTION	VAF, %		Class
				Tissue	Organoid	
1	KRAS	G12D	nonsynonymous SNV	19.05	29.69	Class 1, 2, 3, 4, 5
2	CALR	K159N	nonsynonymous SNV	48.24		Class 3
3	ASXL1	S1503L	nonsynonymous SNV		33.26	Class 4
4	SMAD4	528_529del	frameshift deletion	25.08	53.4	Class 4, 5
5	ABCG2	I196M	nonsynonymous SNV	48.66	51.79	Novel
6	BRD7	277_277del	frameshift deletion	2.98	2.31	Novel
7	CBL	803_803del	frameshift deletion	32.78	31.59	Novel
8	DIS3	A524V	nonsynonymous SNV	30.56		Novel
9	DOT1L	A528V	nonsynonymous SNV	36.13		Novel
10	EGF	E1134fs	frameshift insertion	31.15	29.73	Novel
11	EML4	S978L	nonsynonymous SNV	43.13	43.28	Novel
12	IGF2R	A2459V	nonsynonymous SNV	60.36	73.64	Novel
13	KAT6A	I647M	nonsynonymous SNV	49.71	48.92	Novel
14	KAT6A	1109_1110del	nonframeshift deletion	45.85	45.76	Novel
15	KMT2A	G3131S	nonsynonymous SNV	45.3	48.15	Novel
16	LRP1B	splicing	NA		7.52	Novel
17	MITF	L455I	nonsynonymous SNV	44.29	45.58	Novel
18	TP53		splicing	29.01	50.43	Novel
19	TTF1	A246V	nonsynonymous SNV	50.27	49.7	Novel
20	ZNRF3	V47L	nonsynonymous SNV	49.41	42.96	Novel

CNV

Gene	Status		Class
	Tissue	Organoid	
TYMS	Amp.	Amp.	Class 2
CDKN2A	Del.	Del.	Class 4, 5
CDKN2B		Del.	Class 5
APCDD1	Amp.	Amp.	Novel
NKX2-1		Del.	Novel

TH235

SNV/INDEL

No.	Gene	Amino Acid Change	FUNCTION	VAF, %		Class
				Tissue	Organoid	
1	TP53	R280K	nonsynonymous SNV	35.91		Class 2, 3, 4, 5
2	BRCA2	E3377D	nonsynonymous SNV	49.17	47.54	Class 3, 4, 5
3	CDKN2A	H66R	nonsynonymous SNV	81.16		Class 4
4	MTOR	K449E	nonsynonymous SNV	69.29		Class 4, 5
5	CDH1	E758K	nonsynonymous SNV		30.32	Class 5
6	ABCC3	T1406M	nonsynonymous SNV	45.84	45.77	Novel
7	ARID1B	D172fs	frameshift deletion	29.17	42.5	Novel
8	ATR	G1342V	nonsynonymous SNV	26.01		Novel
9	BRD7	277_277del	frameshift deletion	3.42	3.92	Novel
10	CDH2	F36Y	nonsynonymous SNV	50.98	73.37	Novel
11	CDH5	L394R	nonsynonymous SNV		51.72	Novel
12	CDK8	D366V	nonsynonymous SNV		45.09	Novel
13	DNMT1	L1247V	nonsynonymous SNV		41.2	Novel
14	CUL4A	G611D	nonsynonymous SNV	31.47		Novel
15	FANCA	W22S	nonsynonymous SNV		57.14	Novel
16	FANCC	A325T	nonsynonymous SNV	59.48	67.12	Novel
17	FAT3	V1226E	nonsynonymous SNV	36.49		Novel
18	FGF4	S114R	nonsynonymous SNV	68.96	78.88	Novel
19	FOXP1	H53Q	nonsynonymous SNV	57.63		Novel
20	G6PD	W379C	nonsynonymous SNV	54.11		Novel
21	HDAC2	K11fs	frameshift deletion	1.1		Novel

CNV

Gene	Status		Class
	Tissue	Organoid	
CCND1		Amp.	Class 2, 4, 5
FGFR1		Amp.	Class 2, 4, 5
FGFR1		Amp.	Class 2, 4, 5
CDKN2A		Amp.	Class 4
CEBPA		Amp.	Class 4
EZH2		Del.	Class 4
FGF3		Amp.	Class 4
FGFR3		Del.	Class 4
RSF1	Amp.	Amp.	Class 4
SRSF2	Amp.	Amp.	Class 4
PTPRD	Amp.	Amp.	Class 5
ATR		Amp.	Novel
BCL6		Amp.	Novel
CARD11		Amp.	Novel
CBLB		Del.	Novel
CD274	Amp.	Amp.	Novel
CDH2		Amp.	Novel
CDKN2B		Amp.	Novel
CUL4A		Del.	Novel
DIS3		Del.	Novel
EPHA3		Del.	Novel

22	IGF2R	A2459V	nonsynonymous SNV	70.88		Novel
23	KAT6A	1109_1110del	nonframeshift deletion	32.05	27.41	Novel
24	KDM5A		splicing	28.22	51.43	Novel
25	KMT2A	S215P	nonsynonymous SNV	67.32		Novel
26	KMT2D	P4355S	nonsynonymous SNV	27.86	69.33	Novel
27	KMT2D	Q4085*	stopgain SNV	25	68.75	Novel
28	LTK	S551W	nonsynonymous SNV	25.95		Novel
29	MAP2K7	362_363del	frameshift deletion	10.91	39.09	Novel
30	MSH3	A60delinsAAAP	nonframeshift insertion	12.34		Novel
31	MUS81	L189F	nonsynonymous SNV	48.68	50.69	Novel
32	NCOA3	1243_1246del	nonframeshift deletion	24.16	16.9	Novel
33	NRG1	R391C	nonsynonymous SNV	58.49		Novel
34	PARP3	P271L	nonsynonymous SNV	63.51		Novel
35	PIK3R2	P4S	nonsynonymous SNV	70.73		Novel
36	PKHD1	A1484E	nonsynonymous SNV	25.96	52.52	Novel
37	PTPN11	A50T	nonsynonymous SNV	52.98	61.26	Novel
38	TFRC	T104A	nonsynonymous SNV	62.7	73.66	Novel
39	TMPRSS2	L132F	nonsynonymous SNV	72.44		Novel

EPHA6		Del.	Novel
ERCC5		Del.	Novel
ETV1		Amp.	Novel
FGF14		Del.	Novel
FGF19	Amp.	Amp.	Novel
FGF4	Amp.	Amp.	Novel
FOXL2		Amp.	Novel
FOXP1		Del.	Novel
IDO1		Del.	Novel
IRS2		Del.	Novel
JAK2	Amp.	Amp.	Novel
LAMP1		Del.	Novel
MAD1L1		Amp.	Novel
MCL1		Amp.	Novel
MITF		Del.	Novel
MLLT3		Amp.	Novel
NOTCH2	Amp.	Amp.	Novel
NRG1		Del.	Novel
PAK1	Amp.	Amp.	Novel
PDCD1LG2	Amp.	Amp.	Novel
PIK3CB		Amp.	Novel
PPP2R2A		Del.	Novel
RAD21		Amp.	Novel
RHBDF2		Amp.	Novel
SETBP1		Amp.	Novel

SMAD2		Amp.	Novel
TNKS		Del.	Novel
WRN		Del.	Novel
WT1		Amp.	Novel
XRCC2		Del.	Novel

TH269

SNV/INDEL

No.	Gene	Amino Acid Change	FUNCTION	VAF, %		Class
				Tissue	Organoid	
1	ATM	N1650S	nonsynonymous SNV	55.9	47.18	Class 3, 4, 5
2	GNAQ	M59L	nonsynonymous SNV		0.99	Class 4, 5
3	ABCB1	G187S	nonsynonymous SNV	45.45	43.53	Novel
4	ABL2	P996R	nonsynonymous SNV	59.13	44.83	Novel
5	BRD7	277_277del	frameshift deletion	1.93	3.14	Novel
6	EPHB4	R949Q	nonsynonymous SNV	49.4	53.36	Novel
7	MSH3	55_64del	nonframeshift deletion	24.87	38.8	Novel
8	NCOA3	1243_1244del	nonframeshift deletion	13.19	11.08	Novel
9	NOTCH4	1899_1902del	frameshift deletion		7.47	Novel
10	PARP4	D517N	nonsynonymous SNV	0.94		Novel
11	PIK3R2	P151S	nonsynonymous SNV	58.33	47.73	Novel

CNV

Gene	Status		Class
	Tissue	Organoid	
TERT	Amp.		Class 4
FCGR2B	Del.	Del.	Novel
FGF4	Amp.		Novel
PDPK1	Del.		Novel

TH275

SNV/INDEL

Gene	Amino Acid Change	FUNCTION	VAF, % (Organoid)	Class
ERBB2	R217C	nonsynonymous SNV	50.23	Class 2, 4, 5
BCL2L2	T175M	nonsynonymous SNV	46.71	Novel
BRD7	R96Q	nonsynonymous SNV	43.26	Novel
BRD7	277_277del	frameshift deletion	2.35	Novel
BRIP1	R814C	nonsynonymous SNV	46.92	Novel
EGF	E1134fs	frameshift insertion	31.06	Novel
EPHA7	Q336E	nonsynonymous SNV	47.09	Novel
FGF6	S143N	nonsynonymous SNV	43.85	Novel
GEN1	839_840del	frameshift deletion	42.98	Novel
KAT6A	1109_1110del	nonframeshift deletion	44.01	Novel
KMT2D	S3708R	nonsynonymous SNV	49.8	Novel
LRP6	D1210Y	nonsynonymous SNV	47.51	Novel
MLH1	R217C	nonsynonymous SNV	46.93	Novel
MSH3	A60delinsAAAP	nonframeshift insertion	18.68	Novel
NBN	M553V	nonsynonymous SNV	47.73	Novel
NTRK3	V21F	nonsynonymous SNV	48.64	Novel
POLD1	A171S	nonsynonymous SNV	47.97	Novel
POLD1	R19H	nonsynonymous SNV	47.94	Novel
SETD2	R2098T	nonsynonymous SNV	7.39	Novel

Fusion

Gene	Class
SDHD > SNX9	Novel

UGT1A1	P229Q	nonsynonymous SNV	45.44	Novel

TH276

SNV/INDEL

Gene	Amino Acid Change	FUNCTION	VAF, % (Organoid)	Class
ATM	R3008H	nonsynonymous SNV	72.83	Class 3, 4, 5
NF1	Q400*	stopgain SNV	47.43	Class 4, 5
ERBB4	A1213S	nonsynonymous SNV	63.28	Class 5
POLE	H708D	nonsynonymous SNV	35.6	Class 5
ASNS	V124L	nonsynonymous SNV	0.95	Novel
BRD7	277_277del	frameshift deletion	3.53	Novel
EGF	E1134fs	frameshift insertion	30.92	Novel
ERBB3	K498I	nonsynonymous SNV	73.9	Novel
FANCM		splicing	32.78	Novel
MAP2K4		splicing	35.92	Novel
MAP2K4	D96N	nonsynonymous SNV	41.19	Novel
MAP2K4	G106E	nonsynonymous SNV	43.29	Novel
MAP2K4	R75K	nonsynonymous SNV	34.06	Novel
MDC1	E1415K	nonsynonymous SNV	48.29	Novel
NF1		splicing	48	Novel
NOTCH4	T1582S	nonsynonymous SNV	47.95	Novel
NRG1	S379C	nonsynonymous SNV	47.95	Novel
NTRK3	545_546del	frameshift deletion	28.61	Novel
PKHD1	F2779V	nonsynonymous SNV	36.97	Novel
SDHC	L84F	nonsynonymous SNV	0.78	Novel
TERT	E280K	nonsynonymous SNV	45.89	Novel
VEGFA	K141R	nonsynonymous SNV	60.54	Novel

CNV

Gene	Status (Organoid)	Class
TERT	Amp.	Class 4
BRAF	Amp.	Class 4, 5
CDKN2A	Del.	Class 4, 5
MET	Amp.	Class 4, 5
SMO	Amp.	Class 4, 5
ASNS	Amp.	Class 5
MSH6	Del.	Class 5
ABCB1	Amp.	Novel
CDK6	Amp.	Novel
EML4	Del.	Novel
EPHB4	Amp.	Novel
EPHB6	Amp.	Novel
FOXA1	Amp.	Novel
HGF	Amp.	Novel
HIF1A	Amp.	Novel
NFKBIA	Amp.	Novel
NKX2-1	Amp.	Novel
PIK3CG	Amp.	Novel
TSHR	Amp.	Novel
XRCC2	Amp.	Novel

TH277

SNV/INDEL

Gene	Amino Acid Change	FUNCTION	VAF, % (Organoid)	Class
EGFR	L858R	nonsynonymous SNV	42.4	Class 1, 2, 4, 5
TP53	I255S	nonsynonymous SNV	86.83	Class 2, 3, 4, 5
BCOR	A452T	nonsynonymous SNV	43.21	Class 4
KIT	T304A	nonsynonymous SNV	64.65	Class 4, 5
PTEN	R130G	nonsynonymous SNV	50.27	Class 4, 5
PTEN	G127A	nonsynonymous SNV	35.41	Class 4, 5
APOBEC3B	Q298R	nonsynonymous SNV	47.98	Novel
ARID2	G1709fs	frameshift deletion	25.67	Novel
BRD7	277_277del	frameshift deletion	2.04	Novel
EGF	E1134fs	frameshift insertion	29.97	Novel
EPHB6	F577L	nonsynonymous SNV	26.5	Novel
ERCC3	T215I	nonsynonymous SNV	47.94	Novel
ERCC4	T660I	nonsynonymous SNV	28.39	Novel
ERCC5	D128V	nonsynonymous SNV	58.06	Novel
ESR1	P146Q	nonsynonymous SNV	63.11	Novel
FAT3	R894Q	nonsynonymous SNV	95.7	Novel
FAT3		frameshift substitution	46.18	Novel
FGF10	M204V	nonsynonymous SNV	77.31	Novel
FLCN	E487fs	frameshift deletion	43.45	Novel
GEN1	839_840del	frameshift deletion	29.65	Novel
IGF2R	S1194L	nonsynonymous SNV	35.03	Novel

CNV

Gene	Status (Organoid)	Class
TERT	Amp.	Class 4
RICTOR	Amp.	Class 5
FGF10	Amp.	Novel
IL7R	Amp.	Novel

KMT2A	G3131S	nonsynonymous SNV	48.17	Novel
NCOA3	1243_1244del	nonframeshift deletion	19.63	Novel
POLQ	I2369T	nonsynonymous SNV	30.81	Novel
RHBDF2		splicing	60.76	Novel
SLX4	K99*	stopgain SNV	43.18	Novel
TP53BP1	I1179V	nonsynonymous SNV	45.94	Novel

TH278

SNV/INDEL

Gene	Amino Acid Change	FUNCTION	VAF, % (Organoid)	Class
KRAS	G12C	nonsynonymous SNV	100	Class 1, 2, 3, 4, 5
BARD1	359_365del	nonframeshift deletion	40.11	Class 5
CDH1	L630V	nonsynonymous SNV	29.04	Class 5
AURKA	V84A	nonsynonymous SNV	48.74	Novel
BLM	G512fs	frameshift deletion	0.63	Novel
BRD7	277_277del	frameshift deletion	2.63	Novel
BRD7	Q598R	nonsynonymous SNV	32.79	Novel
CDH5	F156L	nonsynonymous SNV	32.72	Novel
FGFR4	113_114del	frameshift deletion	45.34	Novel
KAT6A	1109_1110del	nonframeshift deletion	30.01	Novel
KAT6A	R1495H	nonsynonymous SNV	64.65	Novel
MSH2	N583S	nonsynonymous SNV	51.28	Novel
NCOA3	1243_1246del	nonframeshift deletion	24.41	Novel
PAK1	183_184del	nonframeshift deletion	1.09	Novel
PKHD1	V3950L	nonsynonymous SNV	43.93	Novel
RET	V706M	nonsynonymous SNV	47.95	Novel
SPEN	2305_2305del	nonframeshift deletion	1.56	Novel
ZNF217	P118S	nonsynonymous SNV	49.65	Novel

CNV

Gene	Status (Organoid)	Class
FGFR1	Amp.	Class 2, 4, 5
FGFR1	Amp.	Class 2, 4, 5
NTRK3	Amp.	Class 4
TERT	Amp.	Class 4
IDH2	Amp.	Class 4, 5
STK11	Del.	Class 4, 5
B2M	Amp.	Novel
BCL2A1	Amp.	Novel
BLM	Amp.	Novel
FANCF	Del.	Novel
FANCI	Amp.	Novel
FGF4	Amp.	Novel
FGF7	Amp.	Novel
FOXL2	Amp.	Novel
IDO1	Amp.	Novel
IGF1R	Amp.	Novel
KAT6A	Amp.	Novel
MAP2K1	Amp.	Novel
MYC	Amp.	Novel
NBN	Amp.	Novel

PML	Amp.	Novel
RAD21	Amp.	Novel
TP53BP1	Amp.	Novel
